# Differences in S/G ratio in natural poplar variants do not predict catalytic depolymerization monomer yields

**DOI:** 10.1038/s41467-019-09986-1

**Published:** 2019-05-02

**Authors:** Eric M. Anderson, Michael L. Stone, Rui Katahira, Michelle Reed, Wellington Muchero, Kelsey J. Ramirez, Gregg T. Beckham, Yuriy Román-Leshkov

**Affiliations:** 1 Department of Chemical Engineering, Massachusetts Institute of Technology, 25 Ames St, Cambridge, MA 02139 USA; 2 National Bioenergy Center, National Renewable Energy Laboratory, 15013 Denver W Pkwy, Golden, CO 80401 USA; 3 Oak Ridge National Laboratory, 1 Bethel Valley Rd, Oak Ridge, TN 37830 USA; 4 The Center for Bioenergy Innovation, Oak Ridge National Laboratory, Oak Ridge, TN 37830 USA

**Keywords:** Biosynthesis, Heterogeneous catalysis, Biofuels

## Abstract

The ratio of syringyl (S) and guaiacyl (G) units in lignin has been regarded as a major factor in determining the maximum monomer yield from lignin depolymerization. This limit arises from the notion that G units are prone to C-C bond formation during lignin biosynthesis, resulting in less ether linkages that generate monomers. This study uses reductive catalytic fractionation (RCF) in flow-through reactors as an analytical tool to depolymerize lignin in poplar with naturally varying S/G ratios, and directly challenges the common conception that the S/G ratio predicts monomer yields. Rather, this work suggests that the plant controls C-O and C-C bond content by regulating monomer transport during lignin biosynthesis. Overall, our results indicate that additional factors beyond the monomeric composition of native lignin are important in developing a fundamental understanding of lignin biosynthesis.

## Abstract (web summary)

The ratio of syringyl (S) and guaiacyl (G) units in lignin has been regarded as a major factor in determining the maximum monomer yield. Here, the authors challenge this common conception using reductive catalytic fractionation in flow-through reactors as an analytical tool to depolymerize lignin in poplar with naturally varying S/G ratios.

## Introduction

The success of second-generation biorefineries hinges on the effective removal and utilization of the lignin fraction of biomass (ranging from 12 to 32 wt% depending on the plant species)^1^. Lignin is a poly-aromatic polymer contained in the cell wall of the plant, which provides structural stability, aids in water transport, and assists in preventing microbial attack of plant cells^2,3^. Accordingly, lignin contributes to the overall recalcitrance of biomass and must be separated before carbohydrates can be successfully and selectively converted into fuels and chemicals^3–6^.

**Fig. 1:**
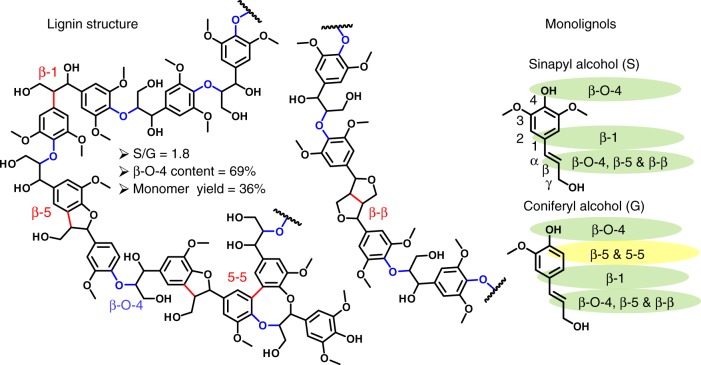
Lignin structure overview. The two primary monomers, sinapyl alcohol and coniferyl alcohol, shown with the different reacting carbons highlighted with the appropriate C–O or C–C bonds that can form. A hypothetical lignin structure is shown with each type of bond as well as the calculated S/G ratio, β-O-4 content, and monomer yield

Lignin is created by the polymerization of three monomers, sinapyl alcohol, coniferyl alcohol, and *p*-coumaryl alcohol, which are synthesized from phenylalanyl and tyrosine in the cytoplasm^7,8^. The plant transports monomers to the cell wall where they undergo free radical coupling reactions creating a variety of C–O and C–C linkages (Fig. 1). This polymerization is mediated by peroxidase and laccase enzymes that form radicals on the phenolic group. By resonance, the radical is shared by the 5, 1, and β position of the monomer^9^ and coupling reactions at any of these positions lead to polymers linked via C–O bonds (β-O-4) and C–C bonds (β-5, 5-5, β-1, and β-β). The β-O-4 bonds are the most abundant and, due to their labile nature, are key for the depolymerization of lignin. The generation of one monomer unit in depolymerization requires cleavage of two β-O-4 bonds, one at each side of the aromatic unit. Therefore, a small number of C–C linkages in the lignin structure can reduce the maximum theoretical monomer content. For example, in the lignin polymer shown in Fig. 1, a β-O-4 content of 69% only yields a maximum monomer yield of 36%. Monomeric units from lignin depolymerization have been shown to be highly valuable as they offer a diverse platform to synthesize chemicals^10–20^ and functional replacements for conventional polymers^21–25^.

Influencing lignin biosynthesis to favor the production of sinapyl alcohol (S-unit) relative to coniferyl alcohol (G-unit) is hypothesized to increase the β-O-4 content in lignin. Sinapyl alcohol has a methoxy group at the 5 position of the aromatic ring, thus preventing the formation of β-5 and 5-5 C–C linkages. Indeed, a higher S/G ratio in lignin has been shown to produce higher monomer yields using reductive catalytic fractionation (RCF) as a depolymerization method, as can be seen in Supplementary Fig. 1, where we compiled a variety of monomer yields found in the literature across a vast range of S/G ratios from different natural and genetically modified feedstocks. Van den Bosch et al.^26^ showed that birch lignin (S/G = 3) subjected to RCF at 523 K produced a monomer yield of 50 C-mol%, while poplar (S/G = 1.5) and a softwood (S/G = 0.05) produced yields of 44 and 21 C-mol%, respectively. Similarly, reports on genetically modified poplar with high or low S/G ratios showed a slight correlation between S/G and monomer yields. Shuai et al. showed that genetically modified poplar (S/G = 58)^27^ produced a monomer yield of 78 wt% when depolymerized using RCF^28^. Interestingly, Parsell et al.^29^ observed a lower monomer yield of 36 wt% for an F5H-modified poplar with an S/G of 2.7 and Luo et al.^30^ observed a yield of 32.5 wt% for a genetically modified low-S poplar (S/G = 0.51). Despite seeing trends across both different species and genetically modified poplar, it is difficult to isolate the effect of S/G ratio on monomer yields from the effects resulting from plant genotype and genetic engineering. We reasoned that the effect of S/G ratio within natural variants of poplar would allow us to better isolate the effect of S/G ratio from other factors.

Although many active stabilization methods have been developed to extract and simultaneously depolymerize lignin into stabile aromatic units^6,31,32^, RCF is effective at achieving near-theoretical lignin monomer yields from β-O-4 bond cleavage. It works through a solvolytic extraction of biomass followed by reductive cleavage of ether linkages in lignin over a redox active catalyst^33^. Typically, RCF is performed in a polar protic solvent^34,35^ with hydrogen gas or a hydrogen donor^36–38^ as a reductant and either Ru^26^, Pd^39^, or Ni^36,40,41^ catalysts at 180–250 °C. RCF depolymerizes lignin by selectively cleaving all β-O-4 linkages within the lignin polymer to produce a stable mixture of monomeric and oligomeric alkyl phenols while preserving carbohydrates as a solid^37,42,43^. Therefore, the distribution of monomeric and oligomeric phenols can be easily mapped to the native lignin structure of the plant.

In this study, we use RCF to investigate the impact of the S/G ratio on the production of monomers using natural poplar variants with S/G contents ranging from 1.41 to 3.60, or percent S content of 58.5% to 78.3% (Table 1)^44^. These five samples capture the range of S/G ratios present in a naturally variant population of over 1000 poplar trees^45^. RCF experiments were performed in flow-through reactors to obtain time- and composition-resolved extraction profiles. Additionally, batch experiments were performed at near complete lignin extraction. The oligomeric fractions were analyzed by heteronuclear single quantum coherence (HSQC) NMR spectroscopy to obtain the time-averaged S/G ratio. Additionally, the oligomers were derivatized by silylation and analyzed by gas chromatography-mass spectrometry (GC-MS) to obtain a qualitative distribution of C–C linkages within the dimeric fraction. These time-resolved data on both the monomeric and oligomeric fractions led to insights on the C–C bonding patterns of S-units that result in a decreased dependency of monomer yields on the S/G ratio than was previously hypothesized in the literature^6^.

**Table 1:** Compositional analysis of poplar natural genetic variants

S/G	% S	% Lignin	% Glucan	% Xylan	% Galactan	% Arabinan	% Mannan	% Acetyl	% Total
1.41	58.5	27.1	39.4	16.8	1.7	0	5.2	4.2	96.7
1.69	62.8	25.1	42.4	14.6	1.8	0	4.4	3.7	95.1
2.35	70.1	23.9	44.7	15.0	1.9	0	5.3	3.9	96.8
3.48	77.7	25.0	39.7	17.3	1.7	0	5.2	4.1	95.9
3.60	78.3	22.6	42.7	17.2	1.8	0	5.1	4.0	96.0

## Results

### Flow-through and batch RCF of poplar natural variants

**Fig. 2:**
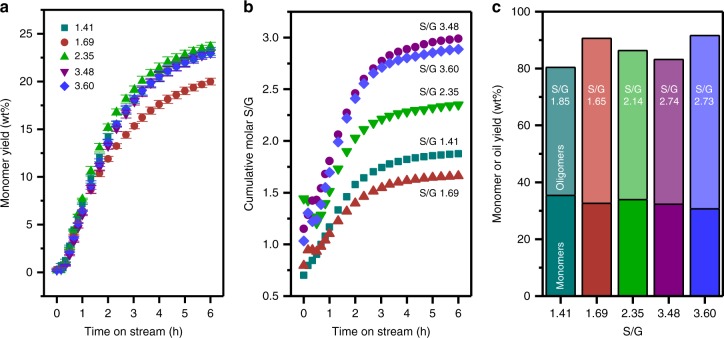
Yields and S/G ratios from flow-through and batch RCF reactions for poplar natural variants. **a** Cumulative monomer yields for all five poplar natural variants (relative to Klason + acid-soluble lignin in the biomass sample). Error bars are 99% confidence intervals for each point generated from a replicate of the experiment on S/G = 1.69 (see Supplementary Fig. 3). **b** The cumulative S/G ratio calculated from the molar ratio of monomers observed from the RCF reaction. The S/G ratios calculated from monomers recovered are shown in text on the bars. Reaction conditions: 0.96–1.15 g of poplar wood (0.26 g lignin), 0.3 g of 15% Ni/C (50/50 SiO_2_), 190 °C both beds, 0.5 mL min^−1^ MeOH, 50 mL min^−1^ H_2_ at 60 bar. **c** Final yield of both monomers and oligomers obtained from a batch reaction. Batch Conditions: 0.96–1.15 g of poplar wood, 0.15 g 15% Ni/C, 50 mL MeOH, 250 °C, 700 RPM, and 30 bar of H_2_ (STP)

RCF was performed with five poplar natural variants in a dual-bed flow-through reactor to test the hypothesis that higher S content should yield more monomers. The solvolysis bed was loaded with whole biomass corresponding to 0.26 g of lignin (Table 1). The reduction bed was loaded with 0.3 g of a 50/50 mix by weight of 15 wt% Ni/C and SiO_2_. This catalyst loading ensured near complete fractionation and reduction of the lignin over the catalyst bed as determined in our previous study^46^. Thus, under these conditions, the rate of monomer production was limited by the rate of solvolysis or lignin extraction as opposed to the rate of catalytic reduction^33^. Both beds were operated at 190 °C and 60 bar with a methanol and hydrogen flow rate of 0.5 and 50 mL min^−1^, respectively. At 6 h on stream, the cumulative monomer yield was approximately 23 wt% from samples with an S/G of 1.41, 2.35, 3.48, and 3.60 while the one with an S/G of 1.69 produced a monomer yield of 20 wt% (Fig. 2a). The yields were calculated relative to total lignin content in the biomass initially, defined as the sum of Klason and acid-soluble lignin contents (lignin content summarized in Supplementary Table 1). The total mass isolated from an RCF run was subjected to extractions with dichloromethane/water to remove sugars. The resulting oil containing both monomers and oligomers, defined as lignin oil, was used as a metric to describe the extent of lignin extraction. Minimal losses of lignin products during DCM extraction were confirmed using gas chromatography-flame ionization detector (GC-FID) analysis of the aqueous phase (Supplementary Fig. 2). Extent of lignin extraction was comparable for all samples with 54, 50, 52, 51, and 53 wt% of lignin extracted for S/G ratios of 1.41, 1.69, 2.35, 3.48, and 3.60, respectively. The S/G ratio of the monomers produced also show only a weak correlation with the S/G ratio of the biomass sample (Fig. 2b).

Importantly, these same trends were observed at experimental conditions resulting in nearly complete lignin extraction. Specifically, batch RCF experiments performed with supercritical methanol resulted in 80–90% extraction and depolymerization of the lignin within the biomass samples. At these high lignin extraction levels, the monomer yields were 34.4, 31.8, 33.0, 31.6, and 30.0 wt% for the same samples with an increasing S/G ranging from 1.41 to 3.60 (Fig. 2c). Notably, the sample with the highest monomer yield had the lowest S/G ratio and the sample with the lowest yield had the highest S/G ratio. The final S/G ratio of the monomers showed similar trends to those at fractional conversion. The invariance in monomer yields with S/G in the native plants is strong evidence that there are differences in the distribution of C–C linked S and G units in the oligomer fraction.

Furthermore, to confirm that the lack of correlation between monomer yields and the S/G ratio was not caused by the RCF method or by limitations due to catalyst choice, we performed batch RCF with a commercial ruthenium on carbon (Ru/C) catalyst as well as thioacidolysis on each sample. Thioacidolysis is a common lignin analytical technique used to measure the relative content of lignin monomers bound by β‐O‐4 linkages^47–49^. These data, summarized in Supplementary Tables 2 and 3 and Supplementary Figs. 4 and 5, show that neither RCF with Ru/C or thioacidolysis yields correlate with the S/G ratio. An important metric to directly compare the depolymerization efficiency of Ru/C with Ni/C is the monomer/oil ratio (i.e. the weight fraction of the lignin oil made up by monomers). While Ru/C generated slightly higher monomer/oil ratios than Ni/C, it showed no trend in monomer/oil ratio across the S/G range. These experiments confirmed that the conclusions drawn from the Ni/C RCF results are valid, despite slight variations in results between different depolymerization methods, and validate the use of flow-through RCF as an analytical technique.

### Characterization of the lignin oils

**Fig. 3:**
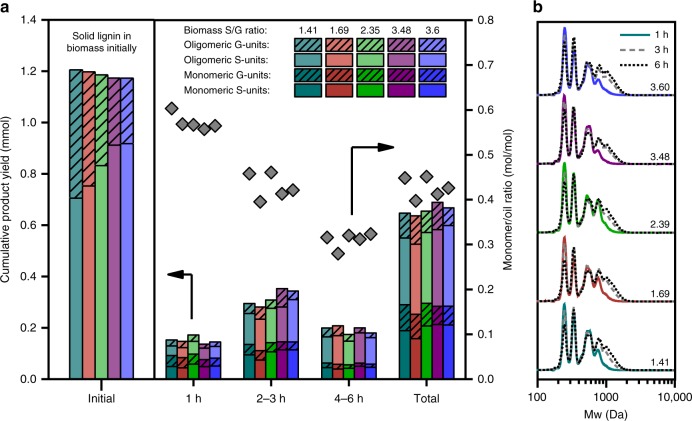
Characterization of the oligomeric fraction of lignin oil produced from flow-through RCF. **a** Mole balance of S and G aromatic units in the monomeric and oligomeric species in lignin oil. The molar monomer-to-oil ratio, which is a measure of lignin fractionation and depolymerization. **b** GPC of the lignin oils from different biomass samples at different RCF extraction times

While the oligomeric fraction of the lignin oil requires more detailed characterization, it offers critical information, such as the molecular weight of lignin fragments, the S/G ratio of the oligomers, and even the identity of C–C linkages in the oligomers. To obtain enough material for analysis, we collected the oil as time-averaged samples at binned times on stream of 1, 2–3, and 4–6 h. The molecular weight of the lignin oil was determined by gel permeation chromatography (GPC), with the samples run at identical lignin oil concentrations to enable comparison. Generally, for all biomass samples, high monomer yields were obtained in earlier time points while high oligomers yields were observed at later time points (Fig. 3a). At 1 h on stream, nearly 60% of the lignin oil consisted of monomers. Subsequently, the monomer-to-oil ratio steadily decreased as the reaction progressed ultimately leading to 70% of the lignin oil consisting of oligomers at 4–6 h. This trend is mirrored in the GPC (Fig. 3b and Mn, Mw, PD summarized in Supplementary Table 4). Initially, all lignin oils contained fragments under 1000 Da, but at later times, a new peak appeared at 1000 Da that extended out to 2000 Da. Additionally, the lowest molecular weight peak associated with monomer content drastically decreased as the reaction progressed.

The S/G ratio of the entire lignin oil was determined with HSQC-NMR spectroscopy by calculating the molar ratio of syringyl to guaiacyl units obtained from integrating the correlation peaks of S_2,6_, S′_2,6_ (oxidized S-unit), and G_2_ (refs. ^50,51^). The correlations used for the S_2,6_ position were 105.2/6.38 and 102.9/6.57 ppm, while 105.9/7.22 ppm was used for S′_2,6_. Total integrals of those peaks corresponded to two correlations (i.e., the 2,6 position in the syringyl aromatic ring). The correlations for the G_2_ position were at 111.1/6.65 and 109.5/6.52 ppm, and their integrals corresponded to one correlation (i.e., the 2-position in the guaiacyl aromatic ring). An example of the HSQC-NMR analysis is shown in Supplementary Fig. 6. Given that the molar quantities of monomeric S and G units in the oil were readily quantified using GC, we were then able to calculate the molar quantities of S and G units in the oligomeric fractions using Eqs. (1) and  (2), respectively. The derivation of these equations is shown in the Supplementary Methods, and relies on the molecular weight of the monomeric units in lignin oil, which we approximated as propyl syringol and propyl guaiacol. A similar method was used to determine the molar quantities of S and G units in the proto-lignin in the solid biomass samples to generate the bars on the left side of Fig. 3a (full details and equations in the Supplementary Methods).

1 $${\mathrm{Mol}}_{{\mathrm{S}},{\mathrm{Olig}}} = \frac{{\mathrm{S}}}{{\mathrm{G}}}\frac{{{\mathrm{Mass}}_{{\mathrm{RCF}}\;{\mathrm{Oil}}}}}{{\left( {\frac{{\mathrm{S}}}{{\mathrm{G}}} \ast {\mathrm{Mw}}_{\mathrm{S}} + {\mathrm{Mw}}_{\mathrm{G}}} \right)}} - {\mathrm{Mol}}_{{\mathrm{S}},{\mathrm{Mon}}},$$

2 $${\mathrm{Mol}}_{{\mathrm{G}},{\mathrm{Olig}}} = \frac{{{\mathrm{Mass}}_{{\mathrm{RCF}}\;{\mathrm{Oil}}}}}{{\left( {\frac{{\mathrm{S}}}{{\mathrm{G}}} \ast{\mathrm{Mw}}_{\mathrm{S}} + {\mathrm{Mw}}_{\mathrm{G}}} \right)}} - {\mathrm{Mol}}_{{\mathrm{G}},{\mathrm{Mon}}}.$$

To best compare the partition of S and G units between monomer and oligomer fractions, we used a mole fraction variable (*x*_S or G_) defined in Eq. (3). It represents the fraction of S (or G) units present in the product mixture that are bound as oligomers relative to the total number of S (or G) units in the mixture (i.e. monomers + oligomers). This value can also be understood as a partition coefficient of S (or G) units into the oligomer fraction. Notably, when the moles of S or G in the oligomer fraction are normalized by the total S or G content in the lignin oil, the trends across all S/G variants collapse to a single trend (Supplementary Table 5).

3 $$x_{{\mathrm{S}}\left( {\mathrm{G}} \right)}\left( {{\mathrm{Moles}}} \right) = \frac{{{\mathrm{Oligomers}}_{{\mathrm{S}}\left( {\mathrm{G}} \right)}}}{{{\mathrm{Oligomers}}_{{\mathrm{S}}\left( {\mathrm{G}} \right)} + {\mathrm{Monomers}}_{{\mathrm{S}}\left( {\mathrm{G}} \right)}}}.$$

At all time points and for all samples, the S units partitioned into the oligomer fraction more than the G units (Supplementary Fig. 7), and both increased their oligomer fraction content over time. Specifically, during the first hour of extraction, the C–C linked oligomers contained 40–50% of all S units and only 30–40% of all G units. At 2–3 h, the S-unit content in the oligomers increased to 50–60% and the G-unit content increased to 40–50%. At 4–6 h, S content reached 60–70%, while G content reached 60–65%. Averaging over all time points shows that 60% of all S units and 50% of all G units existed as C–C linked oligomers. These values directly contradict the expected distribution of S and G units in C–C linked oligomers, since S units should inherently form less C–C bonds during lignin biosynthesis due to a blocked 5 position in the aromatic ring that is responsible for the formation of β-5 and 5-5 linkages.

### Analysis of the dimer fraction of the lignin oils

Given this surprising result, a more detailed analysis of the dimer distribution was necessary to understand what S–S bonds were formed in the oligomer fraction. To study this bonding pattern, the lignin oils from the same five flow-through RCF experiments at 1, 2–3, and 4–6 h on stream were silylated to increase the volatility of the dimeric lignin fragments in the oil. These dimers were then identified by GC-MS and their relative amounts were used to qualitatively understand what C–C linkages were present in the native lignin of poplar with different S/G ratios and how they were released at different extraction times. Given inherent limitations in using GC-MS to analyze lignin dimers, we emphasize that the dimer distribution does not show the complete picture of all C–C linkages in the oligomer fraction. Indeed, this fraction will also always over-predict the amount of total β-1 and β-β linkages in the lignin. These linkages can only form higher C–C linked oligomers when coupled with 5-5 fragment.

**Fig. 4:**
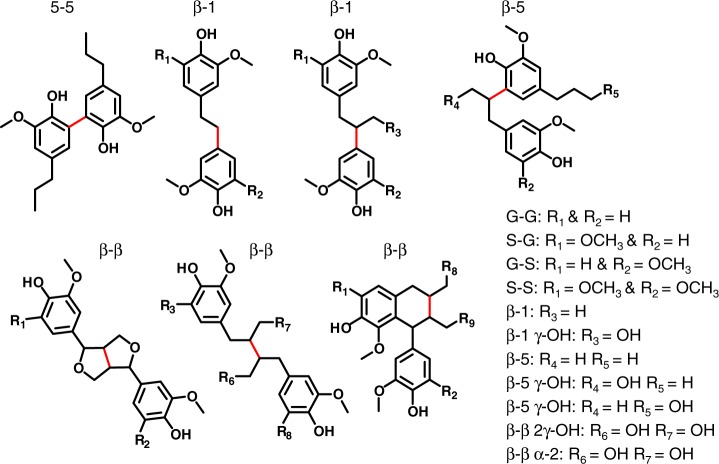
Structures of observable dimers from flow-through RCF. Each dimer shown was identified in the RCF lignin oil using derivatization followed by GC-MS. Red lines indicate the carbon–carbon bond present between two monolignols, which corresponds to the naming convention. Each variation in functionalization (that was identified in the lignin oil) for each dimer is shown in the list on the right

A total of 18 different dimers were identified featuring a variety of S–S, G–G, and S–G units coupled via β-1, β-β, β-5, and 5-5 C–C linkages (Fig. 4, Supplementary Figs. 11–15 for fragmentation analysis of newly identified dimers and Supplementary Figs. 16–28 for fragmentation patterns and citations of previously identified dimers)^35,47,52^. Additional minor variation in dimer composition occurred through deoxygenation of the γ hydroxyl group on the lignin side chain, as well as the formation of a functionalized tetrahydronaphthalene dimer. Lu et al.^53^ previously reported this class of dimers as a decomposition product of the β-β resinol structure while performing derivatization followed by reductive cleavage. This compound is formed by an intramolecular α-condensation reaction with the opposing aromatic ring at the 6 position. Additionally, uncondensed β-β dimers were observed, indicating that the reduction catalyst used in RCF can cleave the ether bonds in the resinol structure.

**Fig. 5:**
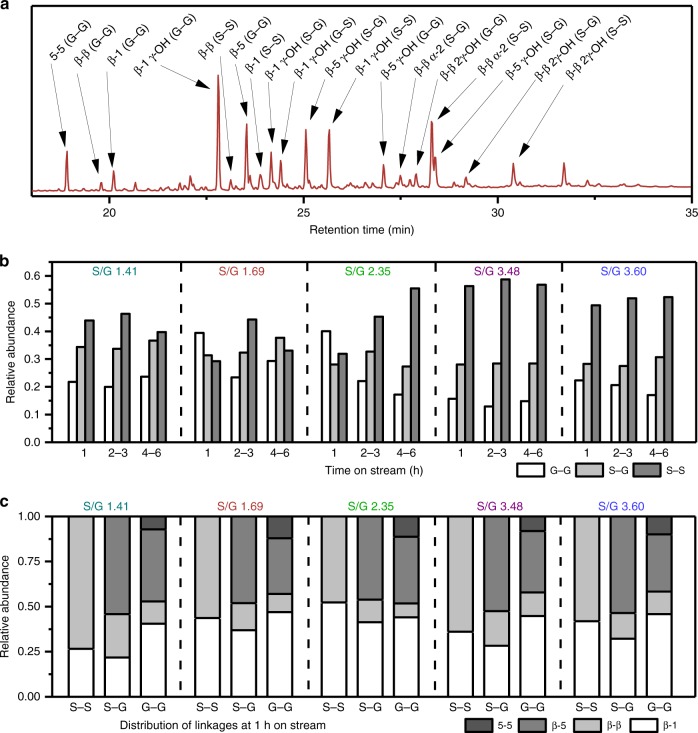
Distribution of C–C linked dimers observed from flow-through RCF reactions. **a** Chromatogram from a representative GC-MS run with a silylated lignin oil (specifically, S/G=1.69 at 1 h on stream). The intensity of the peaks measured in total ion counts. **b** The relative occurrence of the different coupling partners at different times on stream as determined by relative GC-MS total ion count peak areas. **c** The relative distribution of linkage types with in each monomers coupling pair as determined by relative GC-MS total ion count peak areas. Note that all dimer analysis was done on the same samples as those shown in Figs. 2 and 3

Identification and integration of the dimer peaks allowed for qualitative trends of coupling partners and linkage types. While the actual concentrations were not determined with this technique, the relative ratios of peak areas within each individual injection provide a method to qualitatively analyze trends. The trends in S–S, G–G, and S–G dimers formed as a function of time on stream and native S/G ratio are shown in Fig. 5b. Later time points are shown graphically in Supplementary Fig. 8 with the actual values for each linkage shown in Supplementary Table 6. Importantly, for samples with high native S/G values, 50–60% of the dimers exist as S–S pairs, ~30% as mixed S–G dimers, and ~10–20% as G–G pairs. This result suggests that more S–S coupled dimers are formed as the S/G ratio in the native lignin increases.

To understand the abundance of C–C coupled S-units, the distribution of linkage types within each coupling pair was analyzed for each S/G ratio and extraction time. The relative distribution of bonds formed by each coupling pair is shown in Fig. 5c at 1 h on stream (additional time points shown in Supplementary Fig. 8). The most common C–C linked dimer was the β-β linkage, which consisted of approximately 40% of the observed dimers. The S–S linkage was the most common β-β dimer with S–G or G–G dimers making only a small fraction of the observed linkages (Supplementary Fig. 8). The β-1 and β-5 made up on average an additional 50% of the dimer distribution. There was no significant trend observed for β-1 linkages between the different coupling partners. Alternatively, more S–G β-5 linkages were observed than G–G β-5, which ranged from 50% to 70% of all S–G linkages while only 25–40% of G–G linkages were β-5. 5-5 linkages were also observed in a low amount of 2–5%. Some differences in the distribution of C–C linkages were observed over the course of the flow-through extraction. The relative amount of β-β dimers remained unchanged during reaction. The β-1 linkages initially made up a higher percentage of the dimer distribution at early times consisting of 30–40% of the total dimers observed. The occurrence of these linkages steadily decreased at longer extraction times to 20% of the observed dimers at 4–6 h. Conversely, lower amounts of β-5 bonds were observed at ~30% abundance at 1 h which increased to ~50% at later extraction times. The 5-5 linkages were primarily observed at 1 h on stream and are barely detected at 2–3 and 4–6 h.

## Discussion

The differences in monomer yields and lignin composition at different extraction times was unexpected. The initial surge of monomers might be linked to diffusion limitations associated with varying lignin fragment chain lengths. Specifically, the delayed release of larger lignin fragments could be due to incomplete depolymerization, recondensation reactions, or their inherent slower diffusivity through the biomass pores. HSQC-NMR spectroscopy showed little β-O-4 remaining in the lignin oil isolated at 1 h, and slightly more β-O-4 content in the later time points. Additionally, no peaks corresponding to β-O-4 dimers were observed, which previously were observed for incomplete hydrogenolysis^46^. Because of the low β-O-4 content in the RCF products, it is unlikely that incomplete depolymerization of lignin was the cause of the observed trends. Recondensation of lignin occurs through a dehydration step at the α-carbon of monomers, which generates a carbocation that is susceptible to attack from the aromatic ring of other lignin fragments. Indeed, stabilization strategies have been developed to protect this position and reduce recondensation. Lancefield et al. showed that lignin extracted in alcohols resulted in the addition of the alcohol to the α carbon of monomers to prevent condensation reactions^30,54^. Shuai et al.^28^ also showed that formaldehyde treated lignin created a dioxane ring with the hydroxyls at the α and γ positions of monomers, which prevents deleterious dehydrations from occurring. Shuai et al. also showed that monomer yields from a formaldehyde-stabilized lignin were identical to those produced from direct RCF of the same biomass. In this work, we demonstrated similar trends using either Ni/C or commercial Ru/C catalysts, indicating the results obtained were not caused by the type of catalyst used. Therefore, the supercritical RCF experiments performed within this study should indicate a true monomer yield for each natural variant, ruling out recondensation as the cause of the observed trends.

Comparing the data collected in supercritical batch and flow-through experiments in this work further supports the claim that variation in monomer yields due to condensation is unlikely. On average, the monomer-to-oil ratio from supercritical RCF experiments was 0.38, while flow-through RCF had a monomer-to oil ratio of 0.42 implying a similar amount of depolymerization. If lignin condensation had occurred, the monomer-to-oil ratio of 0.6 observed at 1 h on stream would be the expected result from the supercritical RCF runs. Additionally, the only condensation product observed by GC-MS in the dimer region was the intramolecular condensation of the resinol. Taken together, these data indicate that differences in the molecular weight distributions as a function of time are most likely not caused by repolymerization during extraction.

The most probable factor influencing the temporal dependence of monomer yields and types of dimers observed is differences in chain length. Diffusion of lignin in the internal pores of wood particles would allow for small chains to appear early in the extraction while long chains would lag behind. The high occurrence of β-1 linkages observed at early times supports this hypothesis, because β-1 linkages can form from the fragmentation of a growing chain to start a new polymer chain^52^. As these fragments are formed later in the lignin synthesis, they are likely shorter than those chains formed at the beginning of the process. The increase in β-5 bonds over the course of the extraction also supports this hypothesis, since these are C–C linkages formed during chain growth. The high amount of β-β linkages present can only act as starting points to chains because β-β bonds cannot form on a growing chain. The hypothesis of chain diffusion also could explain the initially high monomer yields. The monomer yield will have some dependence on chain length. At two extremes, six 4-unit chains—each with an average of 50% β-O-4 content—will produce a 38–50% yield of monomers (depending on the location of the C–C bonds), while a single chain with 24 units and a 50% β-O-4 content will generate 25% of monomers. This concept was illustrated by Galkin et al.^55^ and is based on the idea that monomer production requires β-O-4 bonds at both the 4- and β-position of the monomer unit in the polymer chain. In short polymers, terminal positions of the chain are important, while in longer chains their contribution is negligible.

**Fig. 6:**
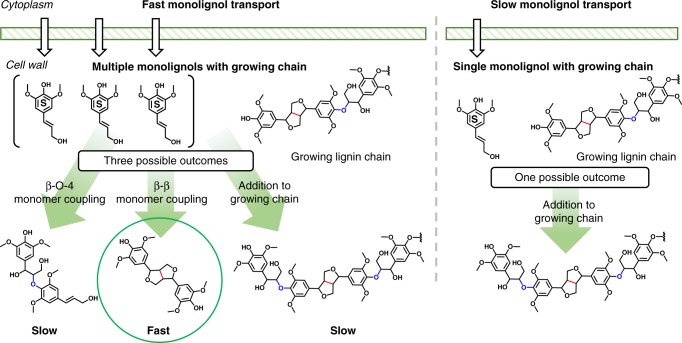
Illustration of monomer concentration influence on bond formation during lignification. In the case of fast monomer transport from the cytoplasm to the cell wall, monomers can couple together to form dimers, or add to growing lignin chains. In the case of slow monomer transport, if an S monomer can only add to a growing chain that already contains a β-β bond, it must form a β-O-4 ether bond

The consistent monomer yields between all of the different natural variants implies there may be some degree of control exerted by the plant that influences the types of linkages formed in the polymerization process. Lignin biosynthesis is dictated by the generation of radicals by peroxidase or laccase enzymes. Radical coupling is fast, and thus the only handle to manipulate the chemistry from a kinetically controlled polymerization is the concentration of the monomers in the cell wall^56^. In vitro experiments to generate dehydrogenase polymers (DHP) have been used to generate synthetic lignin. Batch experiments have been performed with coniferyl and sinapyl alcohol. High monomer concentrations led to the formation of C–C linked dimers with β-5 formed from coniferyl alcohol and β-β dimers from sinapyl alcohol^57,58^. In similar DHP experiments, coniferyl alcohol was slowly added to the reactor maintaining a low concentration. The low concentration led to nearly a 50% yield of β-O-4 linked dimers. Furthermore, when a dialysis membrane was used with both sinapyl and coniferyl alcohol, an insoluble polymer was generated exhibiting similar properties to lignin^59,60^. The importance of monomer delivery rate was shown computationally by van Parijs et al.^61^ who demonstrated that decreasing the influx of monolignols in a lignin polymerization model increased the amount of β-O-4 formation by favoring the growth of longer chains and decreasing dimerization. Density functional theory calculations on the energetics of lignin monomer coupling align with these observations, showing that dimerization of two S units will kinetically favor the formation of β-β bonds^62^. Thus, during lignin biosynthesis, it is plausible that high sinapyl alcohol concentrations relative to the number of growing chains will lead to β-β formation, while a low sinapyl alcohol concentration during lignification would likely lead to formation of β-O-4 in a strictly syringyl polymer. This concept, illustrated in Fig. 6, was also shown experimentally by Stewart et al.^27^ who found that genetically modified poplar with 97.5% S showed a significant increase in levels of β-β linkages (relative to native S-levels in wild-type trees), but an insignificant increase in β-ether levels^27^. Therefore, the consistent monomer yields that we observed for a range of S/G values could be caused by manipulation of monomer concentrations by the plant to control lignin structure and composition leading to similar lignin in different natural variants. Broadly, monomer concentrations during lignification appear to be an important variable to consider when designing lignin for depolymerization in addition to the S/G ratio.

The results of the study contained herein show that there is no correlation between S/G ratio and monomer yields within the range of S/G ratios in the naturally variant poplar population. All data were collected from only five poplar natural variants with differing S/G ratios. Indeed, several other parameters could influence lignin bond formation, including pore structure, plant cell wall microstructure, and lignin carbohydrate linkages^45,63^. In subsequent studies, we will employ a larger population of natural variants to perform multi-variate studies in order to understand to what extent other factors truly influence lignin depolymerization.

The lignin in a series of natural poplar variants with lignin S/G ratios ranging from 1.41 to 3.60 was extracted and depolymerized using RCF in flow-through reactors. Surprisingly there was found to be no correlation between S/G ratio and monomer yields in a flow-through reaction at 50% lignin extraction. Furthermore, when operating at 80–90% lignin extraction, the monomer yields were similar between all poplar samples at approximately 32 wt%. HSQC-NMR spectroscopy, GPC, and silylated GC-MS were performed to understand differences in the high molecular weight fractions of each extracted lignin oil. GPC showed an increase in molecular weight of lignin oil extracted at later times on stream in the flow-through extraction. NMR spectroscopy indicated that these large molecular weight fragments consisted of primarily S lignin units. Analysis of the dimers produced at different times on stream showed an increase in S–G β-5 linkages over time as well as a high amount of S–S β-β linkages throughout the extraction. The similar monomer yields between a wide range of naturally variant wood samples is likely caused by the plants ability to regulate lignification.

## Methods

### Catalyst synthesis

Nickel on carbon (Ni/C) catalysts were synthesized following our previously published wet impregnation procedure^46^. Briefly, to prepare 10 g of 15 wt% catalyst, 7.432 g nickel nitrate hexahydrate (Sigma-Aldrich) was dissolved in 10.2 mL deionized water and added to 8.5 g Darco carbon (Sigma-Aldrich, 100 mesh). After equilibrating at ambient conditions for 16 h and drying for 24 h at 120 °C, the catalyst was thermally reduced in a tube furnace by heating to 450 °C over 1 h and then holding for 2 h at 450 °C under flowing nitrogen (100 mL min^−1^). The catalyst was used in batch reactions without further treatment. For flow reactions, the catalyst required pelletizing to ensure a good flow profile and prevent large pressure drops. Since carbon does not pelletize easily, the 15 wt% Ni/C was mixed 50/50 w/w with SiO_2_ (Sigma-Aldrich, 12 nm) by agitating with a stir bar for 24 h. The resulting physical mixture was pelletized using 6 tons of pressure and sieved to 100–200 mesh.

Ruthenium on carbon (Ru/C) was purchased from Sigma-Aldrich (5 wt% loading) and used as-received.

### Compositional analysis of biomass

Compositional analysis of poplar was performed based on NREL’s Laboratory Analytical Procedure (LAP) at 1/6 the scale^64,65^. To dissolve cellulose and hemicellulose, extracted poplar (0.05 g) was treated with 72 wt% sulfuric acid (0.5 mL) for 1 h at 30 °C. Water was added to decrease the slurry concentration to 4 wt%. The slurry was then heated to 121 °C for 1 h in an autoclave and filtered to yield an acid insoluble lignin-rich fraction. This insoluble fraction was oxidized in air at 575 °C for at least 4 h to determine the ash content. UV/Vis spectroscopy (Thermo Scientific Nanodrop 8000 spectrophotometer) was used to determine acid-soluble lignin content, using absorbance measurements at 240 nm with an extinction coefficient of 2.5. High-performance liquid chromatography (HPLC, Agilent 1100 HPLC) with a refractive index detector at 55 ° C and a Shodex Sugar SP0810 column at 85 °C (0.6 mL min^−1^ of HPLC grade water as the mobile phase) was used for determining sugar content.

Thioacidolysis was performed in duplicate on 2 mg of ground sample, as reported in Harman-Ware et al.^48^.

### Biomass reactivity studies

Batch reactions were performed using a mechanically stirred reactor (Parr, 4560 series, 100 mL) equipped with an overhead stirrer. The reactor was loaded with milled poplar (0.96–1.15 g, 0.075 < *d* < 0.25 mm), catalyst (0.15 g), and methanol (50 mL), then pressurized to 3 MPa with hydrogen gas. While stirring at 700 r.p.m., the reactor was heated to 250 °C over 1 h, held at temperature for 3 h, and quenched with an ice bath. After reaction, the lignin oil fraction was isolated by filtering solids with a 0.2 μm filter, removing methanol under vacuum and performing a dichloromethane (DCM)/water extraction to remove water-soluble sugars. The DCM/water extraction involved dissolving the oil in 20 mL 1:1 v/v DCM and water, recovering the DCM phase, and extracting the water with two additional DCM rinses. Dichloromethane was then removed under vacuum to recover the lignin oil that was then used for product quantification.

Flow-through RCF was performed in a flow-through dual-bed reactor, following our previously published procedure (which also provides in-depth details of the reactor construction)^46^. Briefly, the upstream 1/2″ OD stainless steel solvolysis reactor was packed with 1 g of poplar wood (0.96–1.15 g, 0.075 < *d* < 0.25 mm), held in place with two glass wool plugs, with the remaining void volume filled with 1 mm glass beads. The downstream 1/4″ OD reduction reactor was packed with 0.3 g 15% Ni/C (50/50 SiO_2_, pelletized to 100–200 mesh), also held in place with glass wool plugs, with the remaining void volume filled with glass beads. Methanol was flowed at 0.5 mL min^−1^ into the bottom solvolysis reactor; the solvolysis effluent was mixed with hydrogen (50 mL min^−1^ STP) before flowing through the reduction reactor. The reactor was maintained at 60 bar to ensure the methanol remained a liquid at the operating temperature of 190 °C. Reaction samples were taken every 10 min and analyzed by GC-FID. Liquid oil yields were determined from binned samples which were extracted with DCM/water (3 × 10 mL).

### Lignin monomer quantification

Samples were quantified using gas chromatography (Model 7890 A, Agilent). A 1 μL injection volume was used through a 30 m × 250 μm × 0.25 μm column (DB-1701, Agilent) with a split ratio of 10:1. An inlet temperature of 280 °C and an oven temperature of 50 °C with a 10 °C min^−1^ ramp to 280 °C was used with an overall run time of 29 min. An FID was used to quantify the products, with dimethoxybenzene as an external standard. Allyl syringol (Alfa Aesar, 98%) and propyl guaiacol (Sigma-Aldrich, 99%) were used as calibration standards Additionally, methyl paraben was not quantified in the monomer yield (although it was detected in every sample) as it is a pendent group to lignin attached by an ester linkage.

### Derivatization and GC-MS procedure

Dried lignin oil samples (after DCM extraction) were prepared for derivatization by dissolving in dichloromethane to make a 10 mg mL^−1^ solution. Six hundred microliters of 10 mg mL^−1^ lignin oil solution was added to a 2 mL GC vial followed by 50 μL of pyridine and 100 μL of silylating agent. *N*,*O*-Bis(trimethylsilyl)trifluoroacetamide (BSTFA) with 1% trimethylchlorosilane (Sigma-Aldrich) was used as the silylating agent. Because it reacts readily with water, the BSTFA was ordered in 1 mL ampules and used immediately after opening, being sure to cap the GC-vials immediately after addition of BSTFA. The lignin oil, pyridine, and BSTFA solution was then heated for 20 min at 50 °C before being injected on the GC-MS.

One microliter samples were manually injected with a split ratio of 10:1 and a split flow of 12 mL min^−1^. The inlet temperature was set to 280 °C. The oven was programmed to ramp from 150 to 300 °C at a rate of 5 °C min^−1^ and held at 300 °C for 18 min for a total run time of 49 min. The Agilent Technologies 7820A GC System was equipped with an HP-5ms Ultra Inert 30 m × 250 μm × 0.25 μm column. Products were analyzed using an Agilent 5977B single quadrupole MS detector. A solvent delay of 15 min was used to prevent overloading the detector with the monomers.

GC-MS spectra were analyzed by comparing with dimer structures published in literature and by predicting structures that may be present and comparing them with unknown MS spectra.

### GPC procedure

Oil samples were prepared for GPC by drying 200 μL of 10 mg mL^−1^ lignin oil in DCM solution (which was prepared for derivatization) to isolate 2 mg of lignin oil in a GC vial. This was dissolved in 1 mL of THF to achieve a 2 mg mL^−1^ lignin oil in THF solution.

Three 5 μm PLgel Agilent GPC columns were used in series arranged from larger pore size to smaller pore size from 10^4^to 10^3^ to 50 Å. THF was used as the carrier solvent at a flow rate of 1 mL min^−1^. A Hewlett Packard 1100 series autosampler injected 20 μL injection volumes. The columns were maintained at a constant temperature of 26 °C. The system ran at a pressure of approximately 100 bar, but this was not controlled and was a function of the flow rate and temperature. The eluents were analyzed using a UV diode array detector at a wavelength of 220 nm with a reference wavelength of 360 nm and a 4 nm slit.

A calibration for molecular weight vs residence time was created using a Sigma-Aldrich Fluka analytical polystyrene (low molecular) standard ReadyCal set M(p) 250–70000. The molecular weight vs residence time curve was fit using a polynomial fit, which was used to convert all of the UV response vs residence time data into UV response vs molecular weight data.

### HSQC-2D-NMR procedure

HSQC NMR spectra for extracted lignin oil samples (20 mg dissolved in 0.5 mL deuterated chloroform) were recorded at 25 °C on a Bruker 400 MHz nuclear magnetic resonance (NMR) spectrometer with a 5 mm BBO probe with a Z gradient. The hsqcetgpsi2 pulse program was used for HSQC. Spectra were acquired with a sweep width of 280 ppm in the F1 (^13^C) dimension and 18 ppm in the F2 (^1^H) dimension. A total of 300 scans were performed over 256 increments in the ^13^C dimension with 1024 data points in the ^1^H dimension. An acquisition time of 9.0 ms was used for ^13^C and 71.2 ms was used for ^1^H. A relaxation delay of 1.5 s was used for each spectrum. The solvent peak of chloroform was used as an internal reference (δH 7.24, δC 77.23 ppm).

## Supplementary information

Supplementary Information

Peer Review

Source Data File

## Data Availability

The source data underlying all main text and supplemental figures are provided as a Source Data File.
