# Supplementary material for: Differences in S/G ratio in natural poplar variants do not predict catalytic depolymerization monomer yields
Source: Nat Commun. 2019 May 2;10:2033. doi: 10.1038/s41467-019-09986-1 (PMC6497657; doi:10.1038/s41467-019-09986-1)
Supplement: Supplementary file 1 — Supplementary Information [file 41467_2019_9986_MOESM1_ESM.pdf]

Differences in S/G ratio in natural poplar variants do not predict catalytic depolymerization monomer yields

Eric M. Anderson[a],[ǂ], Michael L. Stone[a],[ǂ], Rui Katahira[b], Michelle Reed[b], Wellington Muchero[c], Kelsey Ramirez[b], Gregg T. Beckham* [b], Yuriy Román-Leshkov*,[a]

(a) Chemical Engineering. Massachusetts Institute of Technology. 25 Ames St. Cambridge, MA 02139
(b) National Renewable Energy Laboratory. 15013 Denver W Pkwy, Golden, CO 80401
(c) Oakridge National Laboratory. 1 Bethel Valley Rd, Oak Ridge, TN 37830
(ǂ) Equal Contributors

Supplementary Information

Supplementary Methods

Calculating the molar S and G content of oligomeric fractions and solid biomass

First, we can define the S/G ratio determined through HSQC-NMR as the following:

$$\frac{\mathrm{S}}{\mathrm{G}} = \frac{\mathrm{Mol_{S,Oil}}}{\mathrm{Mol_{G,Oil}}} \tag{1}$$

With $Mol_{S,Oil}$ referring to the total moles of S units in the lignin oil, including all monomers and oligomers, and $Mol_{G,Oil}$ referring to the total moles of G units in the lignin oil. Next, the average molecular weight of the RCF oil ($MW_{RCF\ oil,AVG}$) can be defined as follows:

$$\mathrm{MW_{RCF\ oil,AVG}} = x_S * \mathrm{MW_S} + x_G * \mathrm{MW_G} \tag{2}$$

With x_S as the mole fraction of S units in the mixture, x_G as the mole fraction of G units, MW_S as the molecular weight of an S unit and MW_G as the molecular weight of a G unit. In RCF oil, an average S unit was assumed to be propyl syringol, with $MW_S = 196\ g/mol$ and an average G unit was assumed to be propyl guaiacol with $MW_G = 166\ g/mol$. Then, x_S and x_G can be written in terms of S/G ratio using the following equations

$$\mathrm{Mol_S} = \frac{\mathrm{S}}{\mathrm{G}} * \mathrm{Mol_G} \tag{3}$$

$$x_S = \frac{\mathrm{Mol_S}}{\mathrm{Mol_S+Mol_G}} = \frac{\frac{\mathrm{S}}{\mathrm{G}}*\mathrm{Mol_G}}{\frac{\mathrm{S}}{\mathrm{G}}*\mathrm{Mol_G}+\mathrm{Mol_G}} = \frac{\frac{\mathrm{S}}{\mathrm{G}}}{\frac{\mathrm{S}}{\mathrm{G}}+1} \tag{4}$$

$$x_G = \frac{\mathrm{Mol_G}}{\mathrm{Mol_S+Mol_G}} = \frac{\mathrm{Mol_G}}{\frac{\mathrm{S}}{\mathrm{G}}*\mathrm{Mol_G}+\mathrm{Mol_G}} = \frac{1}{\frac{\mathrm{S}}{\mathrm{G}}+1} \tag{5}$$

Using Supplementary Equations 4, 5 and 2, $MW_{RCF\ oil,AVG}$ can be calculated with only the S/G ratio from NMR and assumed molecular weights of an S unit and a G unit. Then using the mass of RCF oil from a given reaction, the total moles of S+G units can be calculated, and with S/G ratio, the total moles of G units can be determined

$$\mathrm{Mol_{RCF\ Oil}} = \frac{\mathrm{Mass_{RCF\ Oil}}}{\mathrm{MW_{RCF\ Oil,AVG}}} = \mathrm{Mol_{S,Oil}} + \mathrm{Mol_{G,Oil}} = \frac{\mathrm{S}}{\mathrm{G}} * \mathrm{Mol_{G,Oil}} + \mathrm{Mol_{G,Oil}} \tag{6}$$

Then, the moles of G in the oil can be split into monomer and oligomer fractions

$$\mathrm{Mol_{G,Oil}} = \mathrm{Mol_{G,Mon}} + \mathrm{Mol_{G,Olig}} \tag{7}$$

Since the moles of monomeric G units are known, the moles of oligomeric G units can be determined. Finally, the moles of oligomeric S units can be determined as follows:

$$\mathrm{Mol_{S,Oil}} = \frac{\mathrm{S}}{\mathrm{G}} * \mathrm{Mol_{G,Oil}} = \mathrm{Mol_{S,Mon}} + \mathrm{Mol_{S,Olig}} \tag{8}$$

Using the same set of equations, the moles of S and G units in the solid biomass initially could also be determined, substituting coniferyl alcohol and sinapyl alcohol for the molecular weights of S and G units,

using the mass of the lignin loaded according to the compositional analysis and biomass loading, and using the S/G ratio of the biomass sample as determined by pyrolysis MBMS.

Calculating lignin monomer yields

$$\text{Monomer yield} = \frac{\text{mass of monomers}}{(\text{wt\% Klason+wt\%ASL})*\text{mass of dry biomass}} \qquad (9)$$

Supplementary Tables

Supplementary Table 1: Klason lignin and acid soluble lignin (ASL) used in the total lignin quantification.

S/G	Klason Lignin (wt%)	ASL (wt%)	Total Lignin (wt%)
1.41	22.8±1.2	4.3±0.5	27.1±1.4
1.69	20.9±0.9	4.2±0.2	25.1±0.7
2.35	19.3±0.2	4.6±0.2	23.9±0.1
3.48	19.7±0.4	5.3±0.5	25.0±0.9
3.60	17.3±1.5	5.3±0.4	22.6±1.3

Supplementary Table 2: Thioacidolysis yields for biomass samples. Standard deviation for n=2 reaction replicates shown in parenthesis. Thioacidolysis was performed on 2 mg of ground sample, as reported in Harman-Ware et al.[1]

S/G by Py-MBMS	H (µmol g sample^{-1})	S (µmol g sample^{-1})	G (µmol g sample^{-1})	Total Yield (µmol g sample^{-1})	Measured S/G
1.41	5.7 (±0.0)	330.2 (±6.5)	138.8 (±1.6)	474.7 (±4.9)	2.4 (±0.1)
1.69	5.9 (±0.0)	260.5 (±0.8)	125.9 (±4.5)	392.3 (±5.3)	2.1 (±0.1)
2.35	6.1 (±0.0)	299.0 (±13.0)	106.6 (±1.9)	411.7 (±14.9)	2.8 (±0.1)
3.48	4.8 (±0.0)	353.5 (±9.7)	91.3 (±0.1)	449.6 (±9.9)	3.9 (±0.1)
3.6	5.7 (±0.1)	325.6 (±22.1)	89.5 (±6.8)	420.8 (±29.0)	3.6 (±0.0)

Supplementary Table 3: Comparison of Thioacidolysis and batch RCF yields. Yields are all shown on the same basis – micro-mole of monomer per gram of biomass sample loaded. Measured S/G was calculated based on a molar ratio of monomeric S and G units. The Batch RCF results are the same as those shown in Supplementary Figure 4.

	Ni/C Supercritical Batch RCF Results		Ru/C Supercritical Batch RCF Results		Thioacidolysis Results	
S/G by Py-MBMS	Monomer Yield (μmol g $sample^{-1}$)	Measured S/G	Monomer Yield (μmol g $sample^{-1}$)	Measured S/G	Monomer Yield (μmol g $sample^{-1}$)	Measured S/G
1.41	469.7	1.9	453.7	1.8	474.7 (±4.9)	2.4 (±0.1)
1.69	402.4	1.6	429.6	1.6	392.3 (±5.3)	2.1 (±0.1)
2.35	393.9	2.1	429.7	2.1	411.7 (±14.9)	2.8 (±0.1)
3.48	390.8	2.7	445.1	2.7	449.6 (±9.9)	3.9 (±0.1)
3.6	334.5	2.7	418.4	2.8	420.8 (±29.0)	3.6 (±0.0)

Supplementary Table 4: Mn, Mw, and PD calculated from GPC results (main text figure 3B) for each lignin oil generated in flow-through RCF for each poplar sample at 1 h, 2-3 h and 4-6 h on stream.

S/G Ratio	Time on Stream	Mn	Mw	PD
1.41	1 h	332	448	1.3494
	2-3 h	377	554	1.4695
	4-6 h	406	622	1.532
1.69	1 h	341	476	1.3959
	2-3 h	390	597	1.5308
	4-6 h	418	669	1.6005
2.35	1 h	335	475	1.4179
	2-3 h	378	575	1.5212
	4-6 h	398	620	1.5578
3.48	1 h	314	416	1.3248
	2-3 h	358	543	1.5168
	4-6 h	378	589	1.5582
3.6	1 h	322	442	1.3727
	2-3 h	376	588	1.5638
	4-6 h	402	639	1.5896

Supplementary Table 5: Mole fractions of S in the oligomer fraction of oils generated in flow-through RCF of poplar natural variants. These values were calculated using Supplementary Equations 4 and 5, and S/G ratios generated from integration of HSQC NMR peaks. The data correspond to 1 h, 2-3 h and 4-6 h combined oil samples from reactions performed with the following conditions: 0.96-1.15 g of poplar wood (0.26 g lignin), 0.3 g of 15% Ni/C (50/50 SiO_2), 190°C both beds, 0.5 mL min^{-1} MeOH, 50 mL min^{-1} H_2 at 60 bar.

S/G	1.41		1.69		2.35		3.48		3.6	
Time range	x_S	x_G	x_S	x_G	x_S	x_G	x_S	x_G	x_S	x_G
1 h	43	35	47	38	46	39	48	36	47	36
2-3 h	56	49	62	56	56	47	54	69	59	52
4-6 h	69	66	74	67	69	66	70	61	69	60
Average	58	49	63	54	57	48	58	60	60	49

Supplementary Table 6: Relative abundance of each dimer observed from flow-through RCF of poplar natural variants.

S/G	1.41			1.69			2.35			3.48			3.6		
Time	1 h	2-3 h	4-6 h	1 h	2-3 h	4-6 h	1 h	2-3 h	4-6 h	1 h	2-3 h	4-6 h	1 h	2-3 h	4-6 h
5-5 (G-G)	0.011	0.016	0.008	0.048	0.006	0.008	0.045	0.006	0.008	0.013	0.004	0.012	0.022	0.020	0.006
β-β, resinol (G-G)	0.002	0.002	0.002	0.013	0.002	0.001	0.012	0.001	0.001	0.003	0.001	0.002	0.005	0.007	0.003
β-1, (G-G)	0.012	0.006	0.010	0.025	0.012	0.013	0.025	0.010	0.009	0.004	0.005	0.011	0.007	0.017	0.013
β-1, γ-OH, (G-G)	0.028	0.082	0.048	0.160	0.039	0.046	0.152	0.042	0.038	0.066	0.029	0.035	0.095	0.045	0.025
β-β, resinol, (S-S)	0.005	0.007	0.003	0.015	0.001	0.009	0.020	0.004	0.006	0.005	0.001	0.006	0.008	0.001	0.003
β-5, (G-G)	0.035	0.049	0.057	0.091	0.058	0.082	0.082	0.052	0.058	0.040	0.026	0.036	0.050	0.061	0.030
β-1, (S-S)	0.032	0.025	0.028	0.035	0.021	0.029	0.039	0.036	0.029	0.030	0.028	0.035	0.028	0.045	0.039
β-1, γ-OH, (S-G)	0.011	0.042	0.027	0.066	0.015	0.019	0.070	0.028	0.019	0.046	0.019	0.015	0.049	0.016	0.011
β-1, γ-OH, (G-S)	0.017	0.033	0.043	0.050	0.028	0.034	0.047	0.046	0.041	0.033	0.043	0.032	0.042	0.046	0.032
β-5, γ-OH, (S-G)	0.062	0.077	0.097	0.094	0.054	0.081	0.074	0.083	0.079	0.068	0.062	0.081	0.071	0.071	0.069
β-1, γ-OH, (S-S)	0.082	0.091	0.111	0.093	0.108	0.093	0.128	0.137	0.087	0.173	0.181	0.137	0.179	0.210	0.149
β-5, γ-OH, (G-G)	0.056	0.038	0.035	0.030	0.047	0.085	0.065	0.052	0.065	0.014	0.020	0.029	0.021	0.023	0.038
β-β, α-2, (S-G)	0.011	0.042	0.028	0.022	0.025	0.021	0.023	0.018	0.033	0.030	0.012	0.010	0.021	0.004	0.003
β-β, 2γ-OH, (G-G)	0.028	0.025	0.040	0.026	0.070	0.057	0.019	0.056	0.058	0.018	0.045	0.023	0.023	0.033	0.054
β-β, α-2, (S-S)	0.075	0.228	0.132	0.107	0.118	0.060	0.093	0.097	0.082	0.271	0.154	0.087	0.178	0.068	0.075
β-5, γ-OH, (S-G)	0.134	0.109	0.112	0.056	0.165	0.189	0.055	0.122	0.157	0.080	0.115	0.108	0.080	0.105	0.153
β-β, 2γ-OH, (S-G)	0.038	0.041	0.030	0.025	0.036	0.032	0.012	0.031	0.037	0.024	0.033	0.037	0.019	0.032	0.040
β-β, 2γ-OH, (S-S)	0.361	0.087	0.189	0.043	0.194	0.140	0.039	0.179	0.194	0.085	0.223	0.303	0.101	0.195	0.258

Supplementary Table 7: Monomer formation rates from flow-through RCF experiments

S/G	Rate (g h^{-1})	Rate (mmol h^{-1})
1.41	0.021	0.12
1.69	0.020	0.11
2.35	0.025	0.13
3.48	0.019	0.10
3.6	0.021	0.11

The similarity in production rates implies there is little difference in how the lignin is extracted from the wood particles, regardless of S/G ratio. The monomer production rate is determined by fitting the initial formation rate of monomers from a flow-through extraction.

Supplementary Table 8: Error in compositional analysis based on triplicate measurements.

Identifier	S/G	% Lignin	% Glucan	% Xylan	% Galactan	% Arabinan	% Mannan	% Acetyl	% Total
77	1.41	27.1±1.4	39.4±0.1	16.8±0.1	1.7±0.1	0	5.2±0.4	4.2±0.1	96.7±1.5
507	1.69	25.1±0.7	42.4±0.1	14.6±0.1	1.8±0.1	0	4.4±0.1	3.7±0.1	95.1±0.8
53	2.35	23.9±0.1	44.7±0.2	15.0±0.1	1.9±0.1	0	5.3±0.1	3.9±0.1	96.8±0.2
1672	3.48	25.0±0.9	39.7±0.3	17.3±0.1	1.7±0.1	0	5.2±0.3	4.1±0.1	95.9±0.9
1544	3.60	22.6±1.3	42.7±0.2	17.2±0.1	1.8±0.1	0	5.1±0.1	4.0±0.1	96.0±1.7

Supplementary Table 9: Mn, Mw, and PD calculated from GPC results (Figure S10) for each lignin oil generated in Batch RCF.

S/G Ratio	Mn	Mw	PD
1.41	321	450	1.4019
1.69	331	478	1.4441
2.35	322	448	1.3913
3.48	300	419	1.3967
3.6	313	438	1.3994

Supplementary Figures

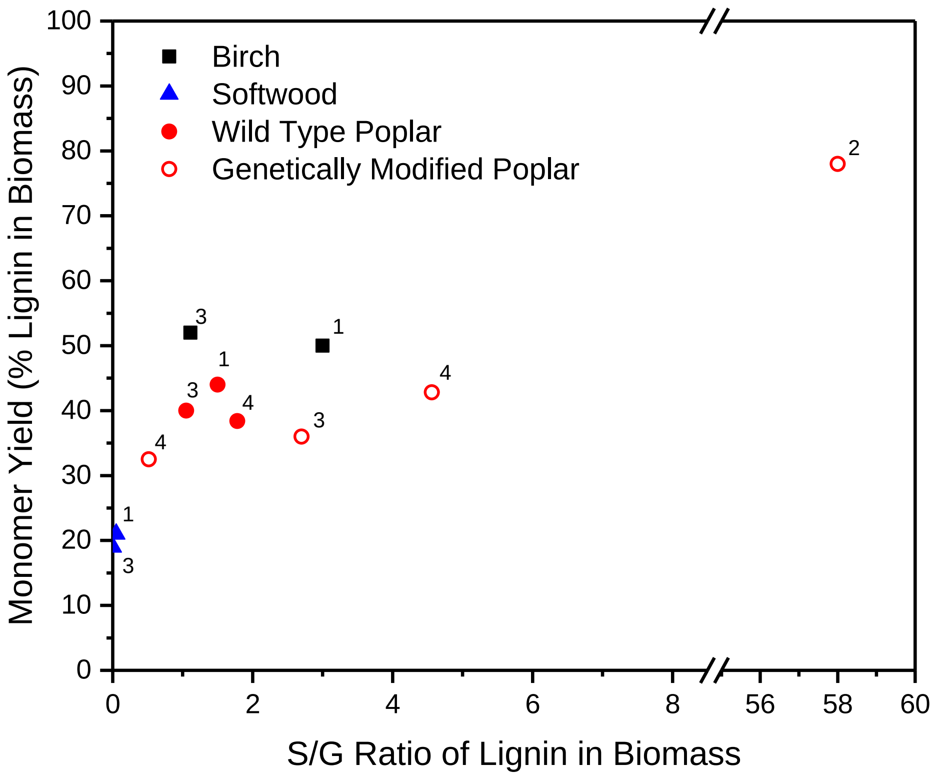

Supplementary Figure 1: Monomer yields reported in the literature from different biomass sources with varying lignin S/G ratios. Citations: 1. Van den Bosch, S. et al. Energy & Environmental Science ***2015,*** *8 (6), 1748-1763., 2. Shuai, L. et al. Science* ***2016,*** *354 (6310), 329-333., 3. Parsell, T. et al. Green Chemistry* ***2015,*** *17 (3), 1492-1499., and 4. Luo, H.; Abu-Omar, M. M Green Chemistry* ***2018,*** *20 (3), 745-753.*

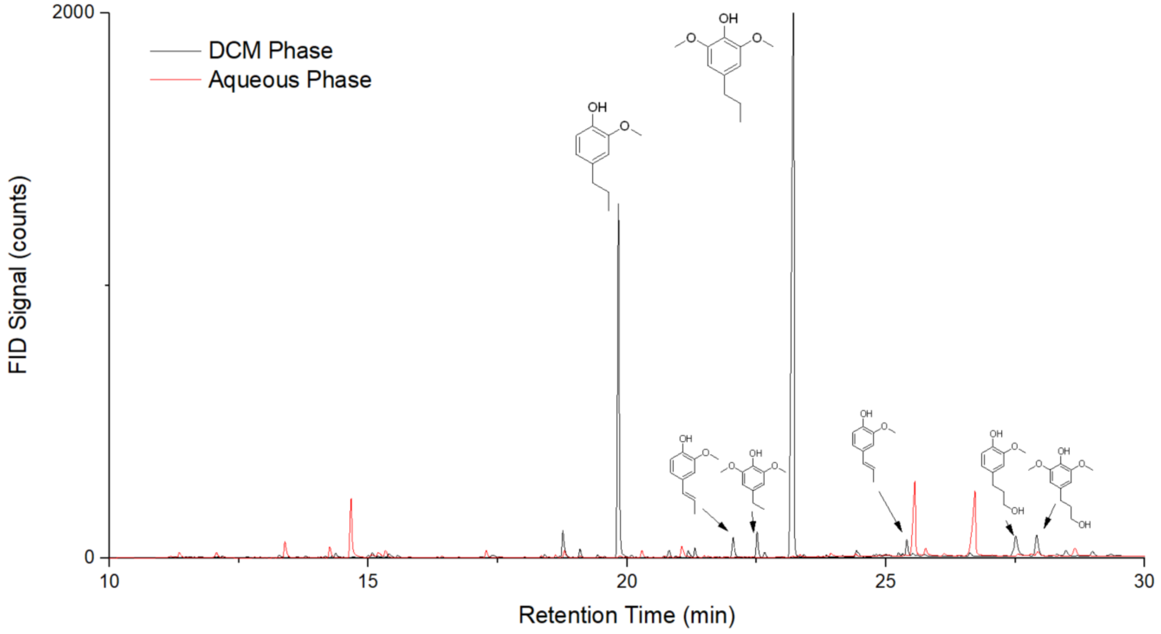

Supplementary Figure 2: GC-FID performed on the aqueous phase after DCM extraction on a supercritical batch RCF experiment with Ru/C. The minor peaks corresponding to lignin monomer compounds in the aqueous phase correspond to ~1.3% monomer yield (versus 32% in the DCM phase)

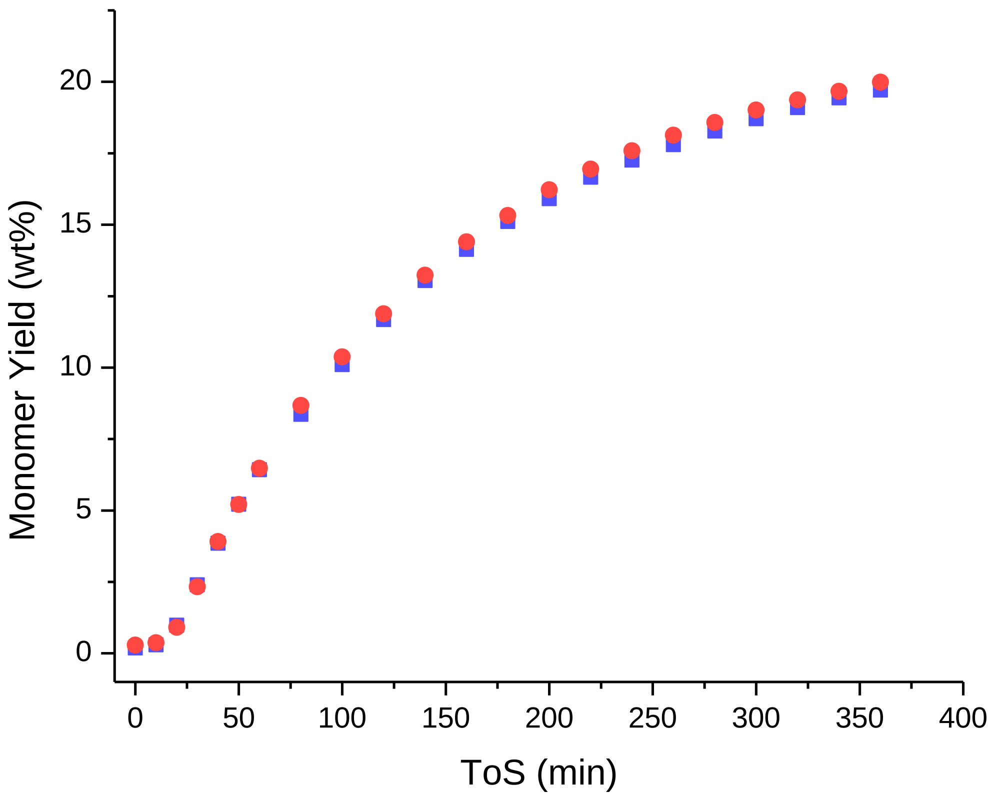

Supplementary Figure 3: Overlay of replicate runs with poplar with an S/G ratio of 1.69. Reaction conditions: 1.04 g of poplar wood (0.26 lignin), 0.3 g of 15% Ni/C (50/50 SiO_2), 190ºC both beds, 0.5 mL min^{-1} MeOH, 50 mL min^{-1} H_2 at 60 bar.

Error bars for flow-through experiments were calculated from the replicate run of sample 1.69. A 99% confidence interval was applied for each point. A percent error was then calculated for each point and applied to all S/G samples in the flow- through experiments.

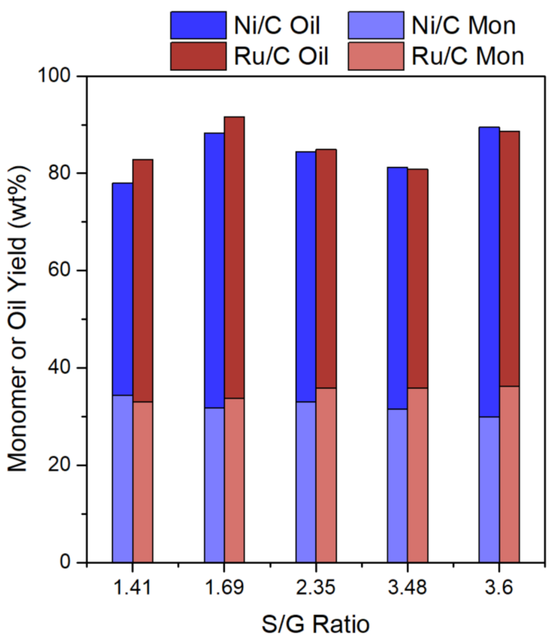

Supplementary Figure 4. Comparison of batch RCF results using Ni/C and Ru/C catalysts. Standard deviations of 1.66% and 0.98% were calculated for the monomer yield and the oil yield, respectively, based on a replicate using Ru/C on the S/G = 3.6 sample. Yields were calculated relative to initial lignin content present in the biomass (Klason + acid soluble). Reaction conditions: 0.96-1.15 g of poplar wood (0.26g lignin), 0.15 g 15 wt% Ni/C or 0.2 g 5 wt% Ru/C, 50 mL MeOH, 250°C, 700 RPM and 30 bar of H_2 *(STP).*

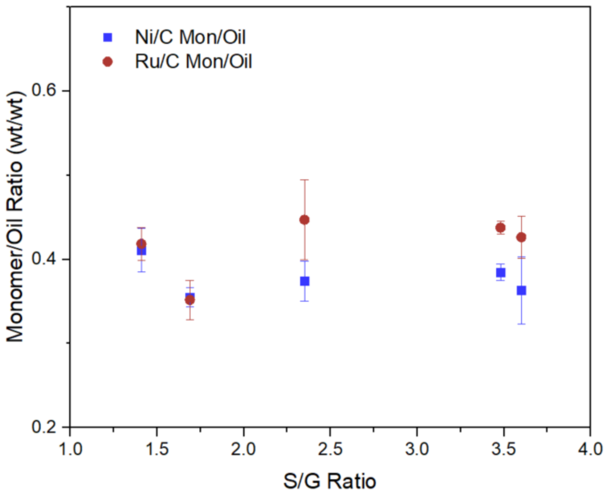

Supplementary Figure 5. Comparison of batch RCF results using Ni/C and Ru/C catalysts. Monomer/oil ratios were calculated as the ratio of the total lignin monomer yield to the total lignin oil yield on a weight basis. Error bars show one standard deviation and were generated using triplicates performed on a scaled-down batch reactor system, using identical reaction conditions to those shown in Supplementary Figure 2 with loadings downscaled to 3/5 of the original.

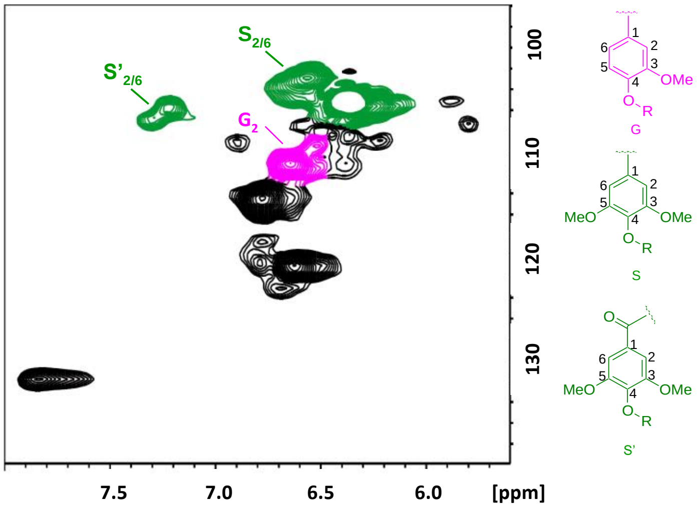

Supplementary Figure 6. Expanded HSQC NMR spectrum of lignin oil from S/G = 2.35 at 2-3 h on stream in the aromatic region. G: guaiacyl, S: syringyl, and S': syringyl with Cα=O

Integration of each peak is 2676 of S2/6, 50 of S'2/6 and 385 of G2. Total integral of S2/6 and S'2/6 corresponded to two correlations, while integral of G2 corresponded to one correlation. Therefore, based on the integration values, S/G ratio was calculated as follows.

$[(S2/6 + S'2/6)/2]/G2 = [(2676 + 50)/2]/385 = 3.54$

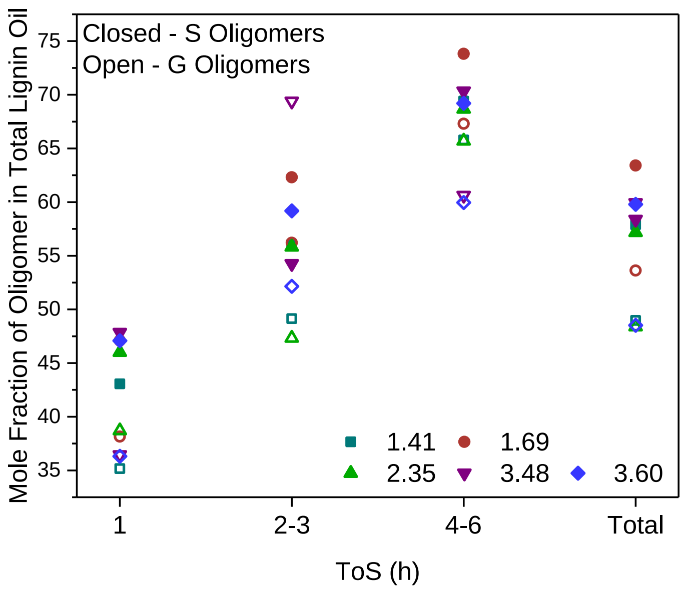

Supplementary Figure 7: Oligomer mole fractions calculated from the mole balance determined from monomer yields and HSQC NMR. The mole fraction displays how much of S units or G units are contained in C-C bonds in the lignin extracted from flow-through RCF experiments. Generally, it can be seen that more S-units (closed shapes) are partitioning into the oligomer fraction than G-units (open shapes) are partitioning into the oligomer fraction. This is true for nearly all time points and nearly all biomass samples.

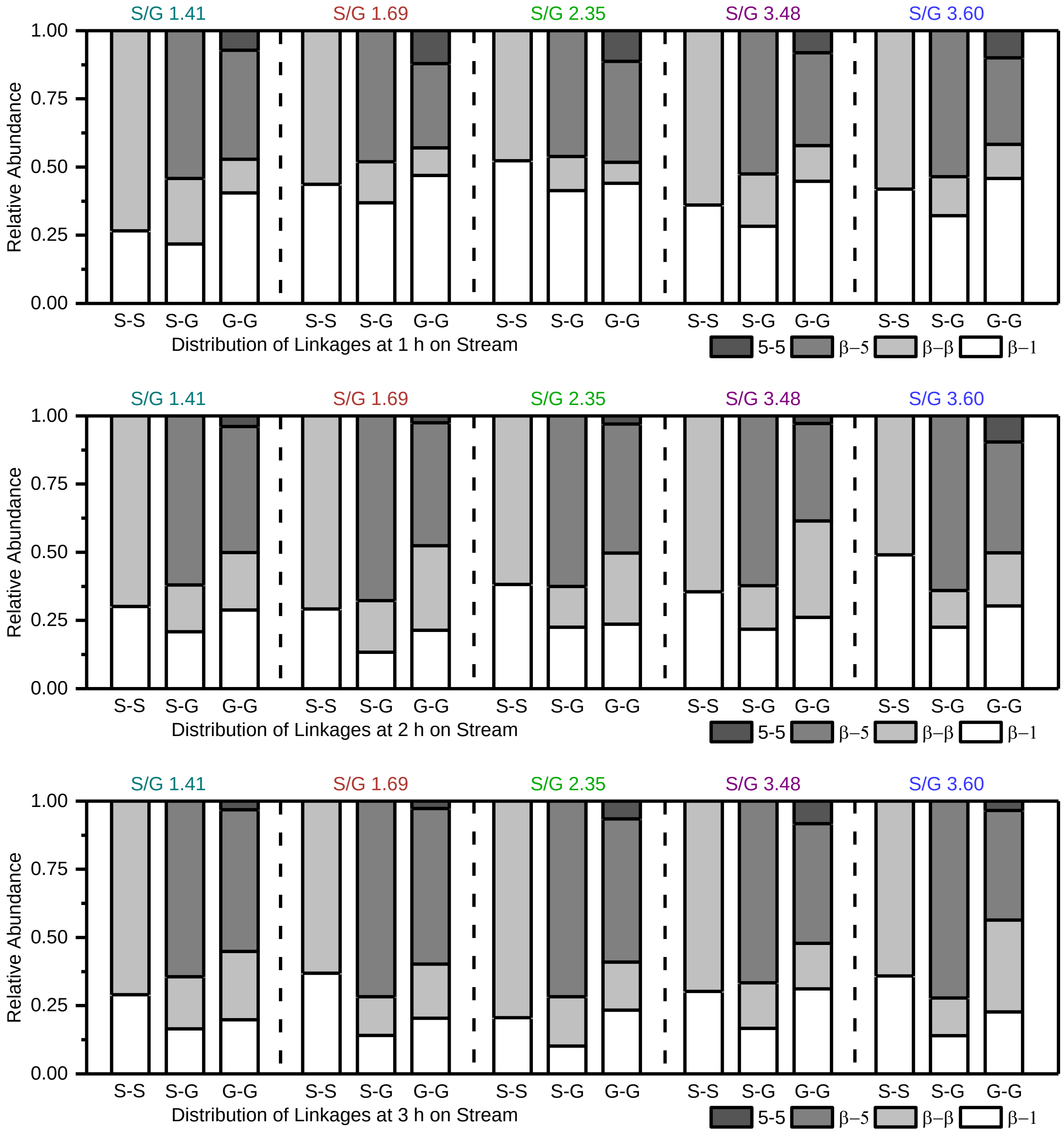

Supplementary Figure 8: relative abundance of C-C dimers from flow-through RCF runs. Reaction conditions: 0.96-1.15 g of poplar wood (0.26 lignin), 0.3 g of 15% Ni/C (50/50 SiO_2), 190ºC both beds, 0.5 mL min^{-1} MeOH, 50 mL min^{-1} H_2 at 60 bar.

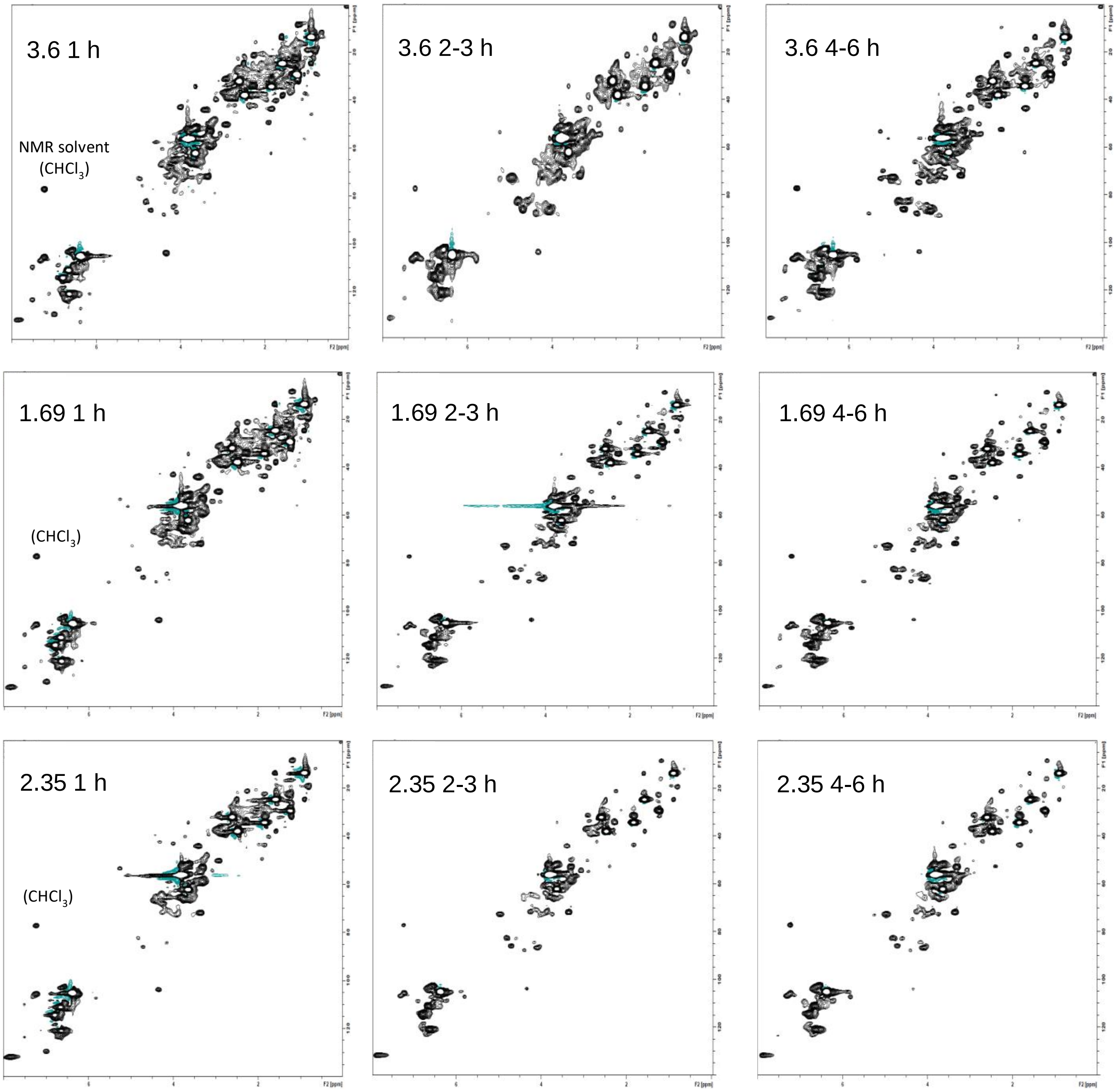

3.6 1 h
NMR solvent
($CHCl_3$)
3.6 2-3 h
3.6 4-6 h
1.69 1 h
($CHCl_3$)
1.69 2-3 h
1.69 4-6 h
2.35 1 h
($CHCl_3$)
2.35 2-3 h
2.35 4-6 h

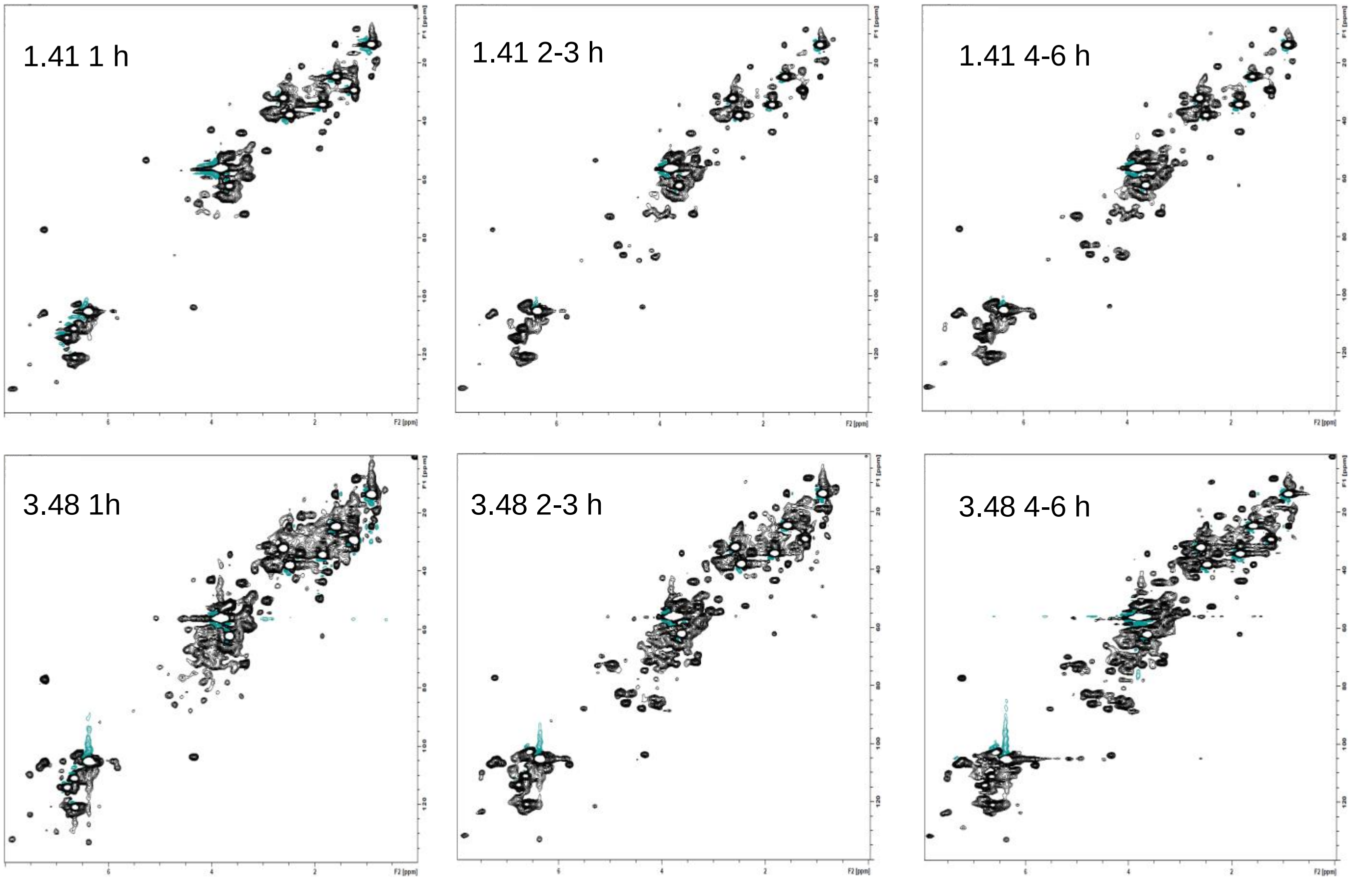

Supplementary Figure 9: HSQC NMR of flow-through RCF runs at 1 h, 2-3 h and 4-6 h on stream. Reaction conditions: 0.96-1.15 g of poplar wood (0.26 lignin), 0.3 g of 15% Ni/C (50/50 SiO_2), 190°C both beds, 0.5 mL min^{-1} MeOH, 50 mL min^{-1} H_2 at 60 bar.

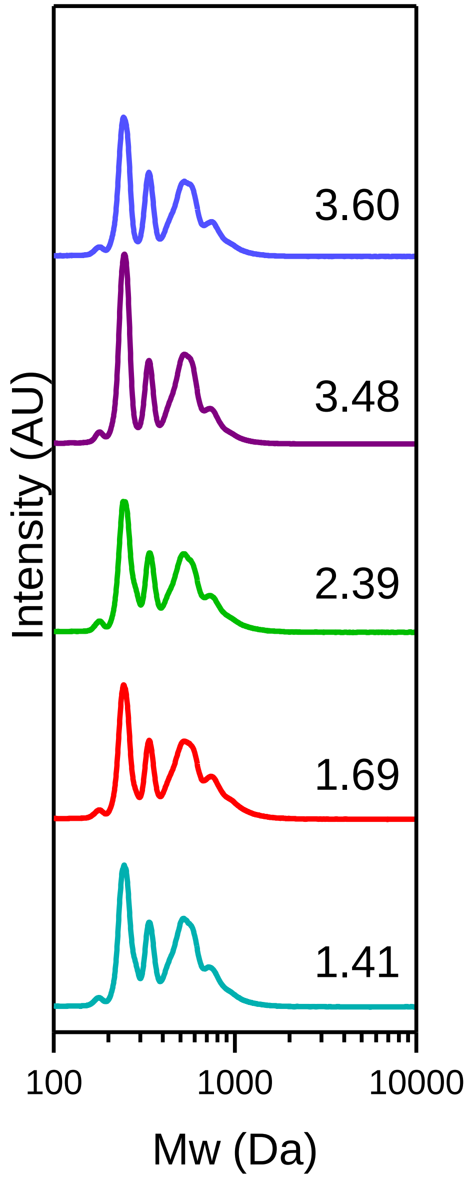

Supplementary Figure 10: GPC for batch RCF runs with natural poplar variants. Batch Conditions: 0.96-1.15 g of poplar wood, 0.15 g 15% Ni/C, 50 mL MeOH, 250ºC, 700 RPM and 30 bar of H_2 (STP).

MS spectra and fragmentation analysis for previously unidentified dimers

RT = 19.801 min β-β (G-G resinol)

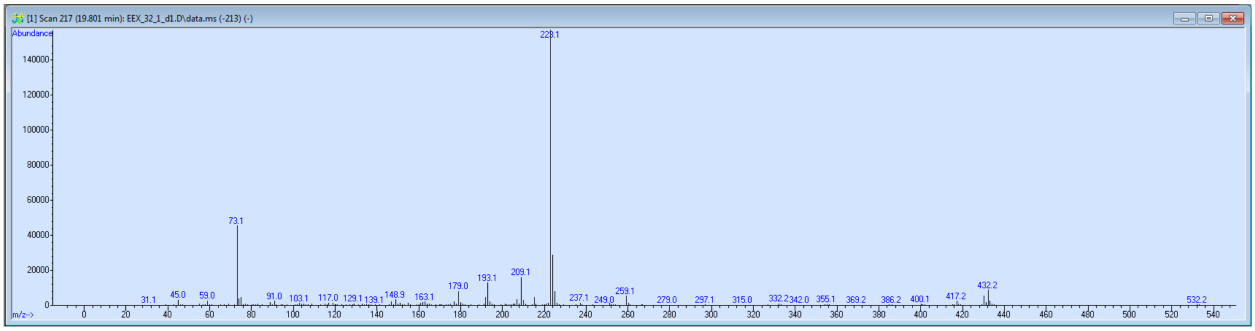

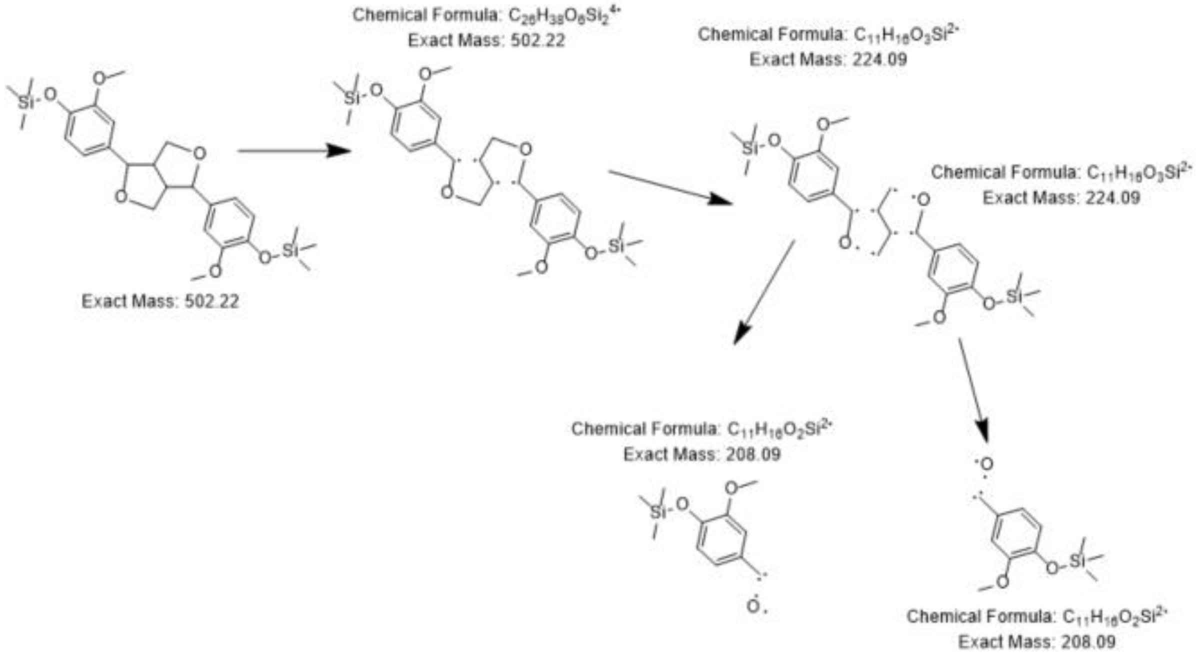

Supplementary Figure 11: Mass spectrum and fragmentation pattern analysis for β-β (G-G resinol) dimer.

RT = 27.490 min β-β a-2 (S-G)

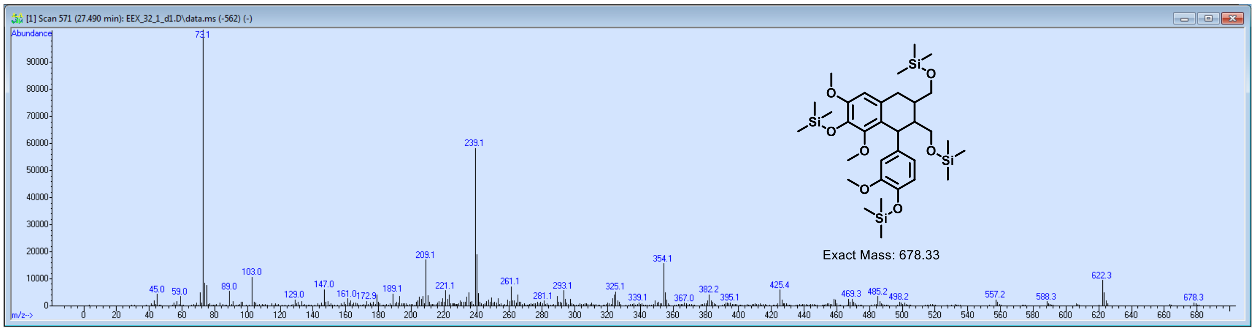

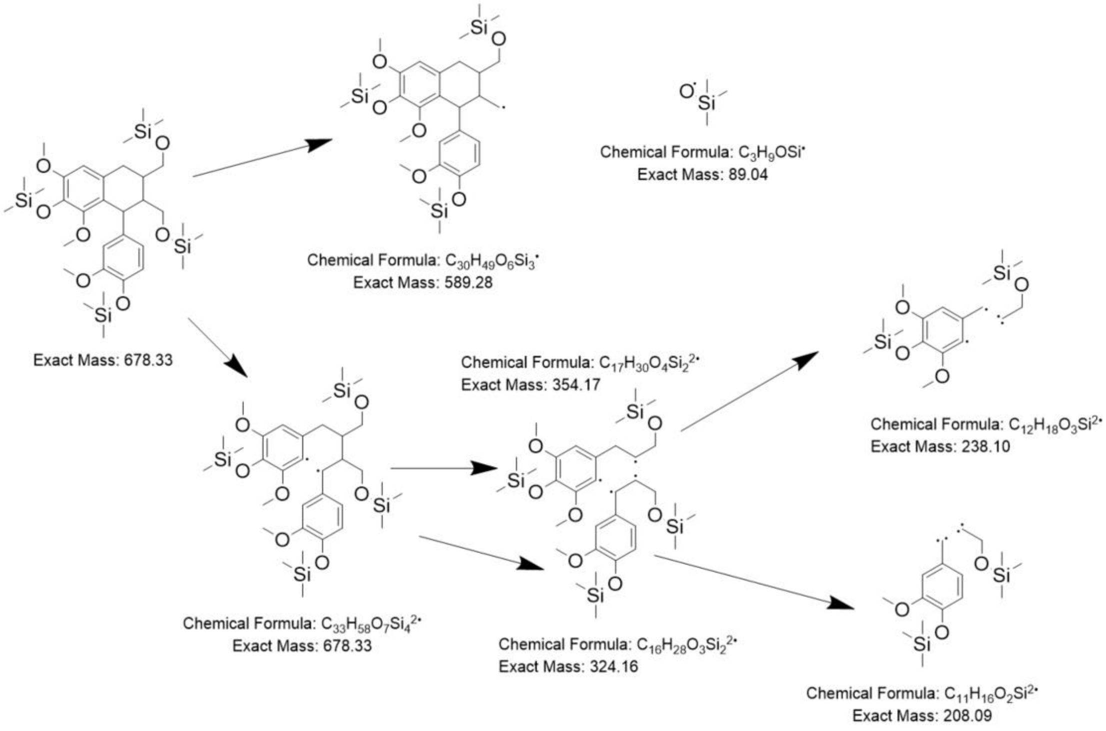

Supplementary Figure 12: Mass spectrum and fragmentation pattern analysis for β-β a-2 (S-G) dimer.

RT = 27.903 min β-β (G-G)

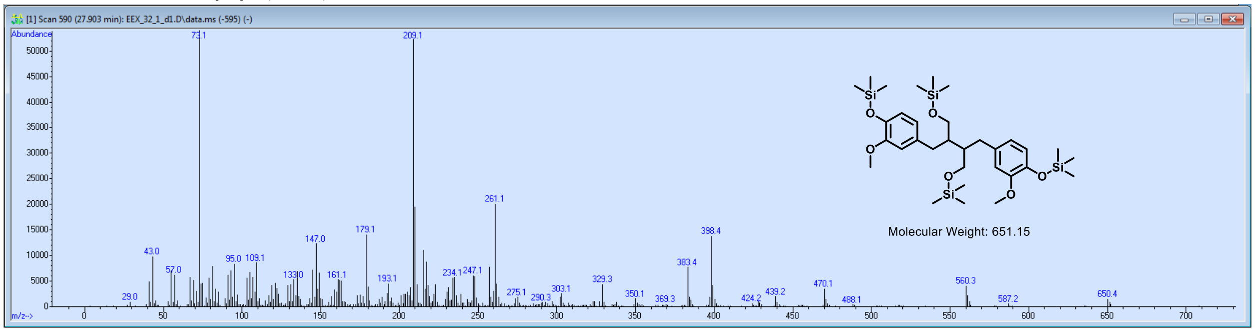

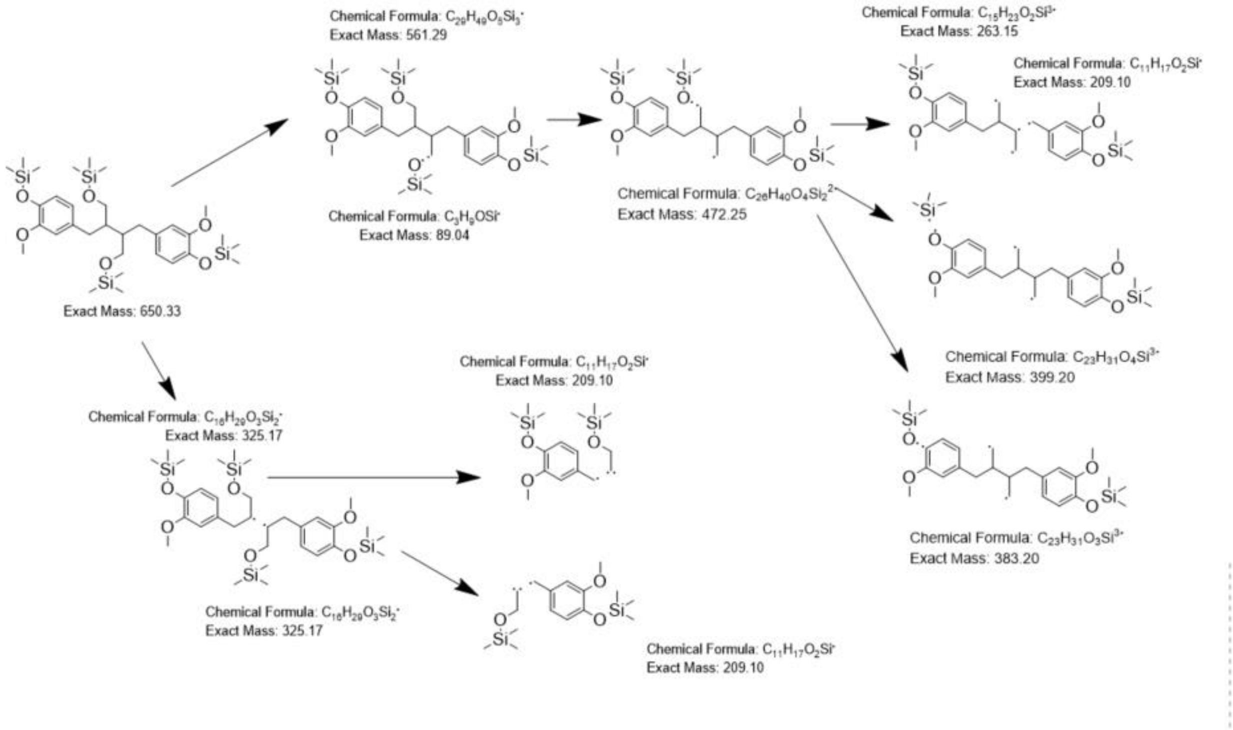

Supplementary Figure 13: Mass spectrum and fragmentation pattern analysis for β-β (G-G) dimer.

RT = 28.294 min β-β (S-S A-2)

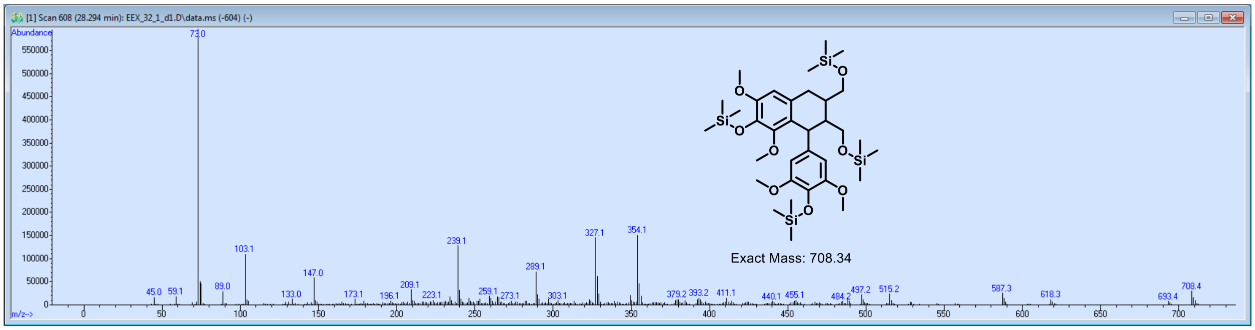

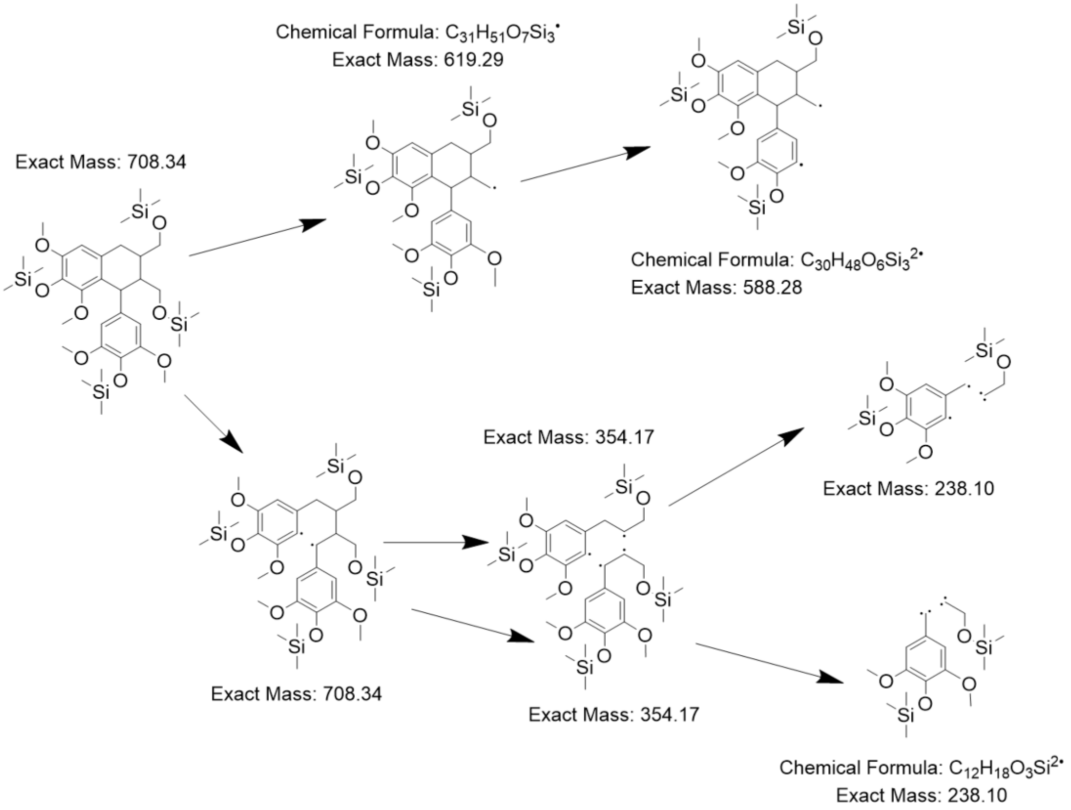

Supplementary Figure 14: Mass spectrum and fragmentation pattern analysis for β-β (G-G) dimer.

RT = 29.184 min β-β (S-G)

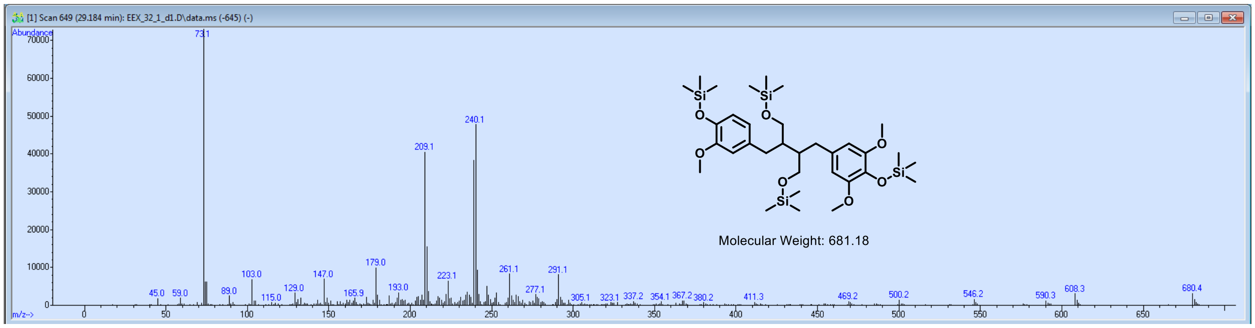

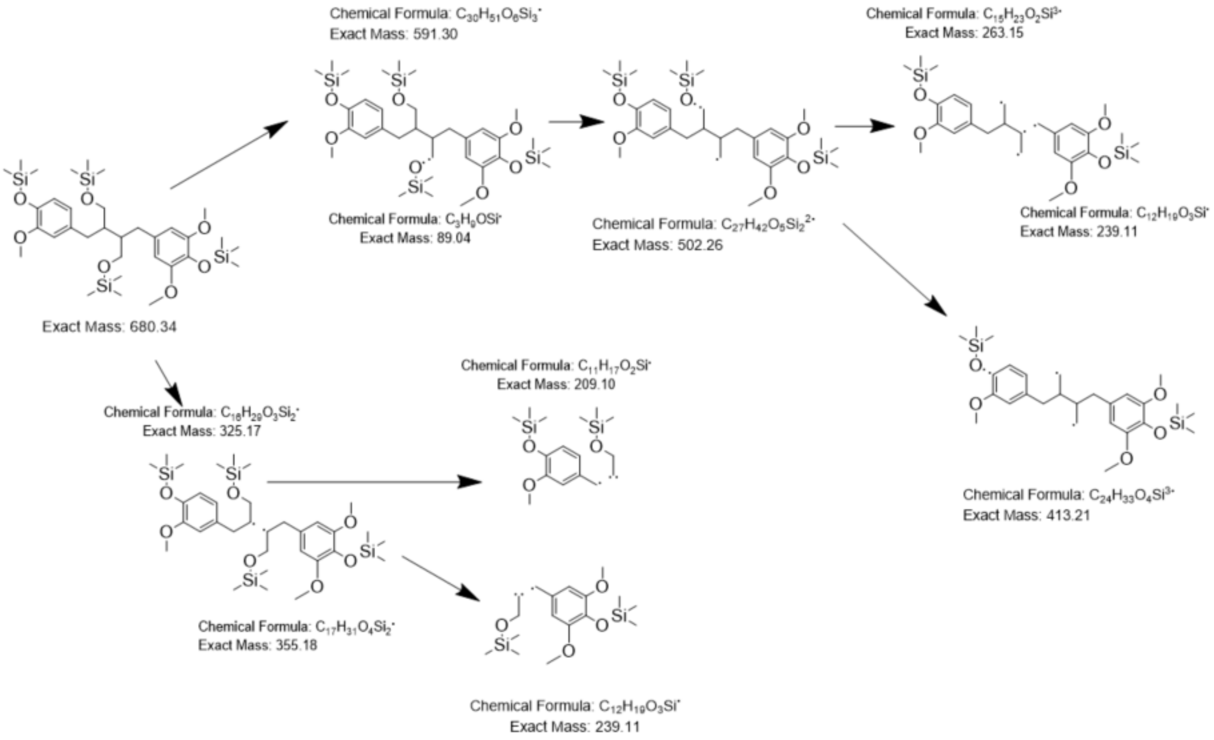

Supplementary Figure 15: Mass spectrum and fragmentation pattern analysis for β-β (S-G) dimer.

MS spectra, dimer structure and corresponding literature citation for previously identified dimers

RT = 18.91 min

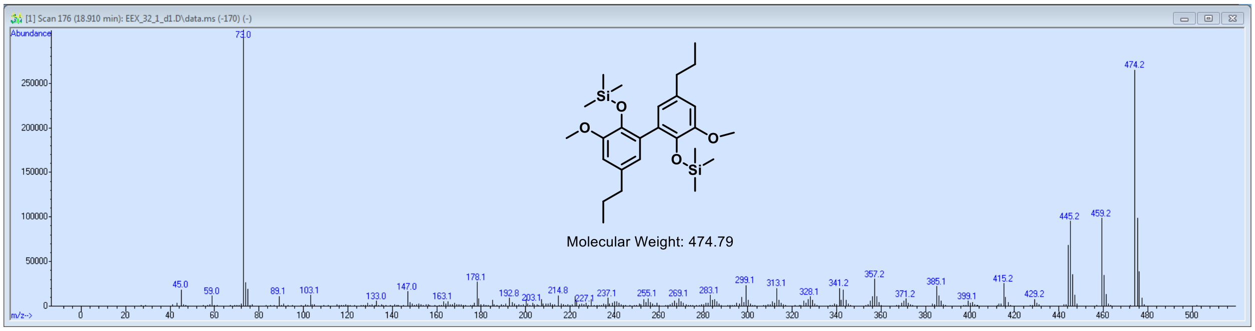

Supplementary Figure 16: Fragmentation pattern for 5-5 (G-G) dimer.[2,3]

RT = 20.105 min

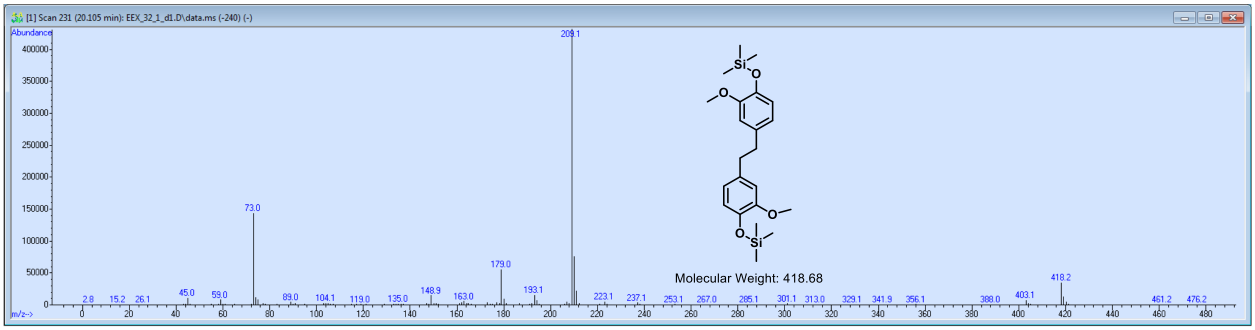

Supplementary Figure 17: Fragmentation pattern for β-1 (G-G) dimer.[2,3]

RT = 22.820 min

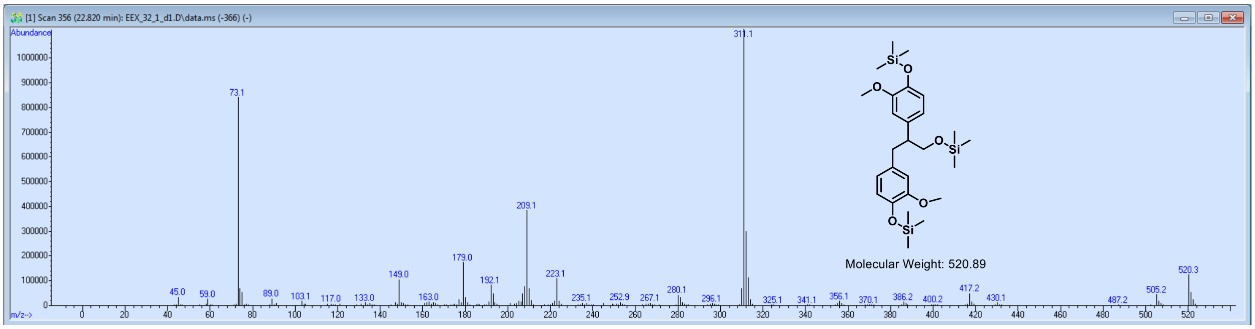

Supplementary Figure 18: Fragmentation pattern for β-1, gamma OH (G-G) dimer.[2,3]

RT = 23.124 min

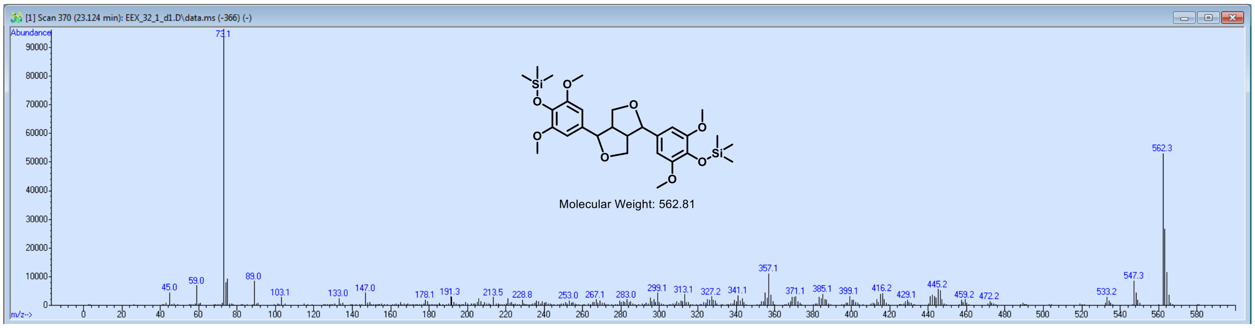

Supplementary Figure 19: Fragmentation pattern for β-β (S-S resinol) dimer.[4]

RT = 23.537 min

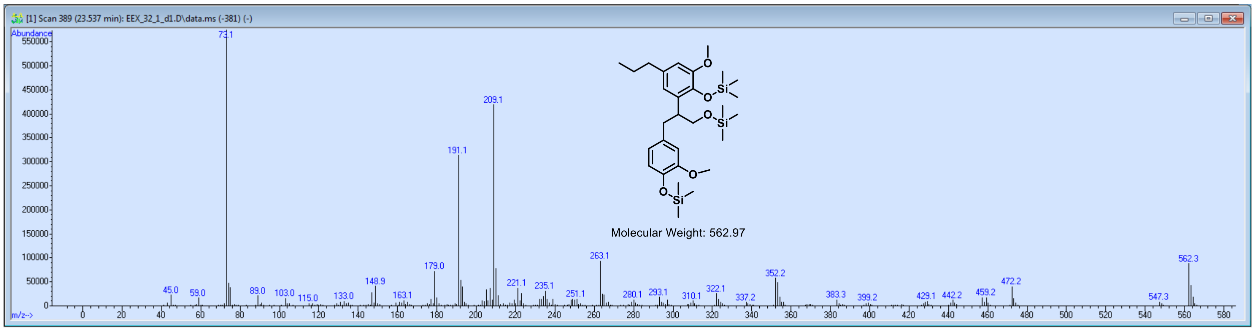

Supplementary Figure 20: Fragmentation pattern for β-5 (G-G) dimer. [2,3]

RT = 23.884 min

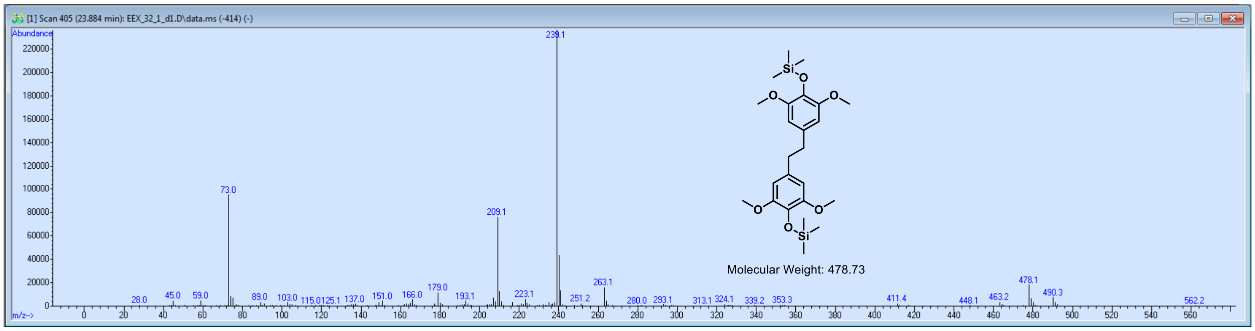

Supplementary Figure 21: Fragmentation pattern for β-1 (S-S) dimer.[3]

RT = 24.167 min

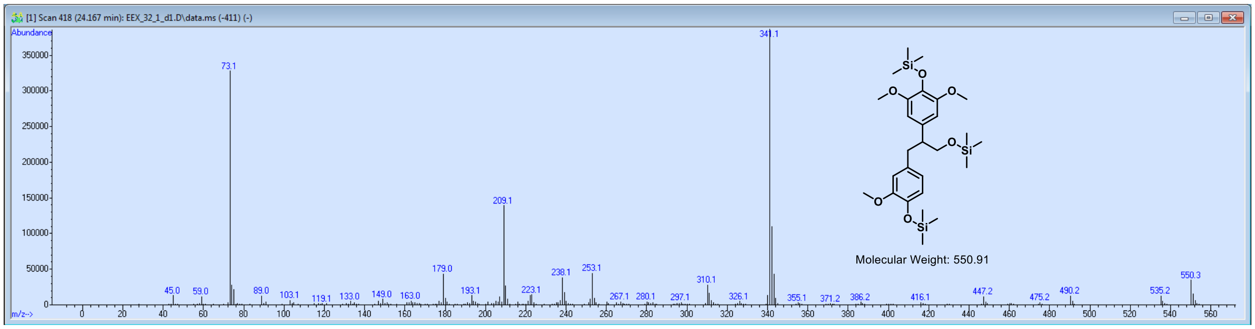

Supplementary Figure 22: Fragmentation pattern for β-1 gamma OH (S-G) dimer.[3]

RT = 24.406 min

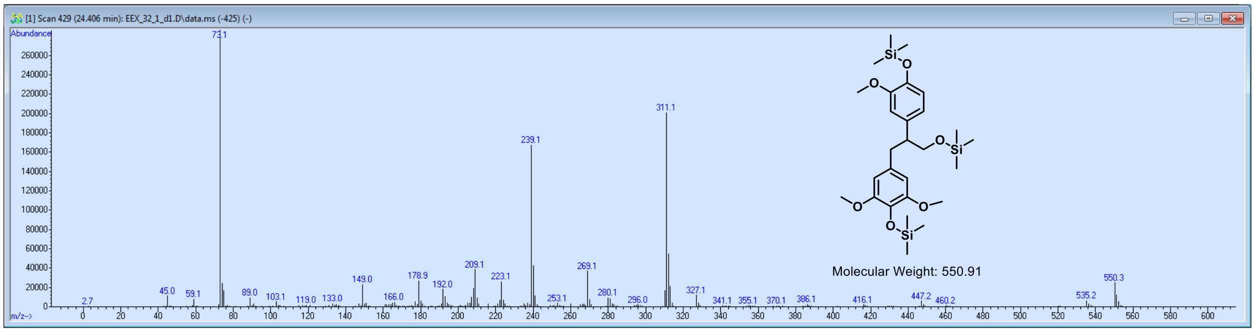

Supplementary Figure 23: Fragmentation pattern for β-1 gamma OH (G-S) dimer.[3]

RT = 25.057 min

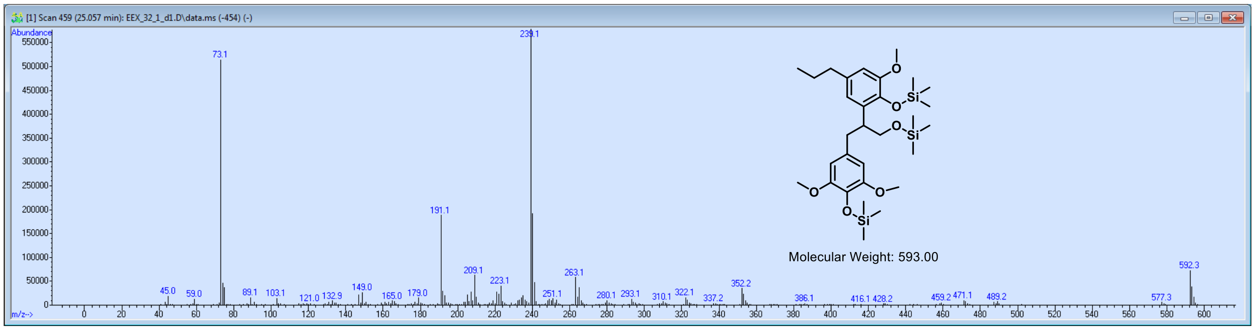

Supplementary Figure 24: Fragmentation pattern for β-5 gamma OH (S-G) dimer.[3]

RT = 25.665 min

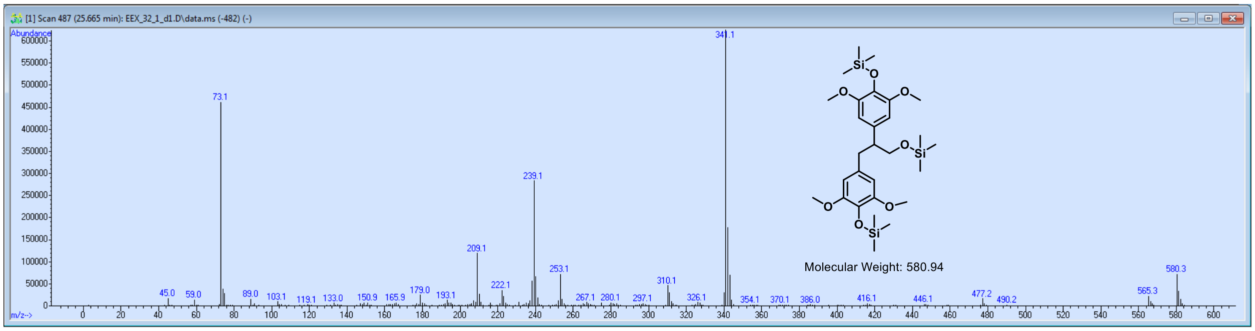

Supplementary Figure 25: Fragmentation pattern for β-1 gamma OH (S-S) dimer.[3,4]

RT = 27.055 min

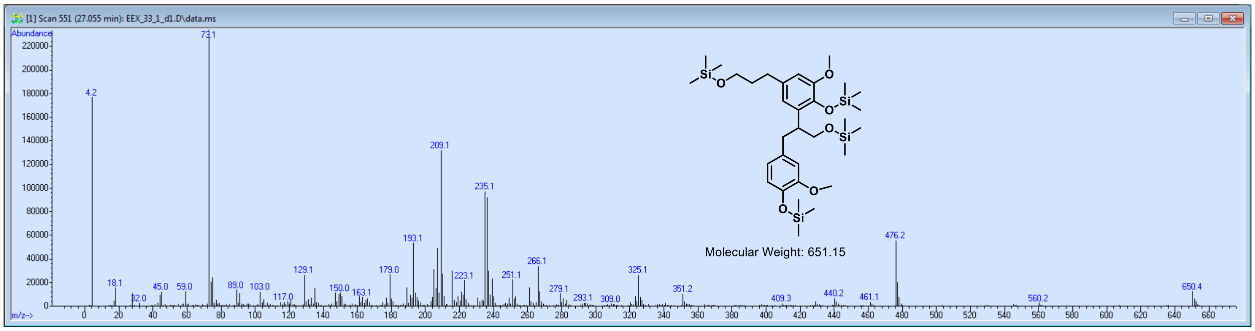

Supplementary Figure 26: Fragmentation pattern for β-5 gamma OH (G-G) dimer.[4]

RT = 28.380 min

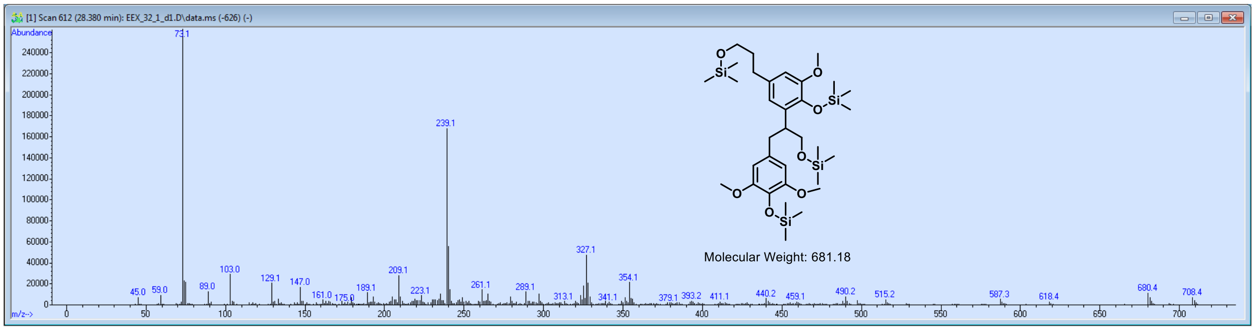

Supplementary Figure 27: Fragmentation pattern for β-5 gamma OH (S-G) dimer.[4]

RT = 30.401 min

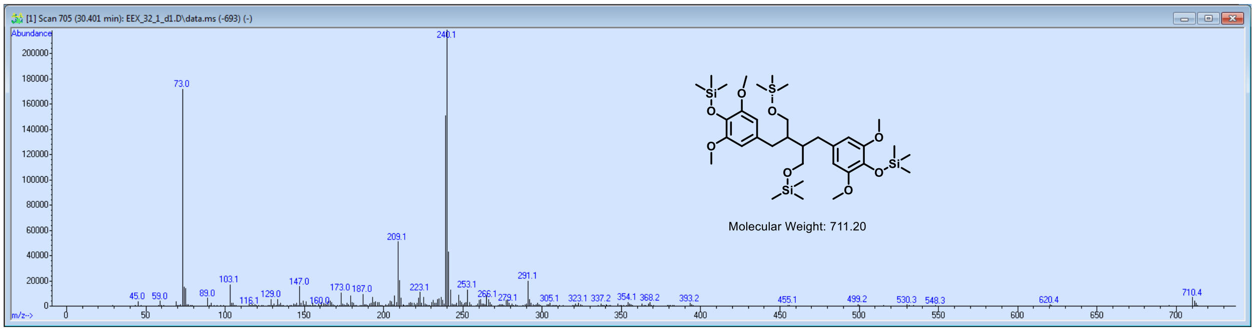

Supplementary Figure 28: Fragmentation pattern for β- β (S-S) dimer.[4]

Supplementary References

1 Harman-Ware, A. E. *et al.* A thioacidolysis method tailored for higher-throughput quantitative analysis of lignin monomers. *Biotechnol. J.* **11**, 1268-1273 (2016).

2 Yue, F., Lu, F., Regner, M., Sun, R. & Ralph, J. Lignin-Derived Thioacidolysis Dimers: Reevaluation, New Products, Authentication, and Quantification. *ChemSusChem* **10**, 830-835 (2017).

3 Heitner, C., Dimmel, D. & Schmidt, J. *Lignin and lignans: advances in chemistry*. (CRC press, 2016).

4 Schutyser, W. *et al.* Influence of bio-based solvents on the catalytic reductive fractionation of birch wood. *Green Chemistry* **17**, 5035-5045 (2015).
