# Supplementary material for: Differences in S/G ratio in natural poplar variants do not predict catalytic depolymerization monomer yields
Source: Nat Commun. 2019 May 2;10:2033. doi: 10.1038/s41467-019-09986-1 (PMC6497657; doi:10.1038/s41467-019-09986-1)
Supplement: Supplementary file 2 — Peer Review [file 41467_2019_9986_MOESM2_ESM.pdf]

Reviewers' comments:

Reviewer #1 (Remarks to the Author):

The manuscript by Roman-Leshkov, Beckham, and co-workers is an intriguing and original investigation on the influence of lignin's S/G ratio, its relation to lignin structure, and implications for lignin depolymerization. The authors exploit their recently developed flow-through RCF system as an analytical tool to study lignin depolymerization in function of reaction time. Surprisingly, the S/G ratio does not seem to correlate with lignin monomer yields. Rather the monomer concentration in the cell wall is presented as an important parameter that governs inter-unit bond distribution during lignin biosynthesis. In my opinion, these findings provide new insights, of general interest to the lignin community, and can be published in Nature communications. However, I feel the following revisions are required to support the conclusions.

• In my opinion, presenting the phenolic distribution as S/G-ratio gives the reader an (erroneous) impression that the distribution differences are very large. Presenting the phenolic distribution as %S and %G is more appropriate in my opinion, and shows that the S-content only differs between 58% and 78% of the total phenolics.

• To strengthen the conclusion, it would be good if the authors could investigate a larger variation in the phenolic distribution, and maybe a larger sample set. Sample 4 and 5 have the same %S.

• I appreciate the approach of using RCF as an analytical technique, though I strongly suggest to benchmark the results with a generally accepted analytical technique, for instance thioacidolysis.

• The authors mention in the discussion that incomplete depolymerization nor recondensation can explain the observed trends. Considering recondensation, I feel that this should be demonstrated experimentally and not merely based on literature. The absence of condensation could be demonstrated by showing that other (noble metal) catalysts result in similar monomer yields and distributions (for example in batch runs). This would demonstrate that the obtained yield is indeed a theoretical maximum yield.

• The conclusions seem to align with conclusions from ref. 26. I suggest the authors to include this in the discussion.

• Related to the previous comment, I feel the following paper should be included/discussed.
van Parijs FR, Morreel K, Ralph J, Boerjan W, Merks RM. Modeling lignin polymerization. I. Simulation model of dehydrogenation polymers. Plant Physiol. 2010;153(3):1332-44. Herein, the flux of monomers during lignin biosynthesis is also appointed as an important factor in the distribution between C-C and C-O-C interunit linkages, though the results are based on model insights in combination with data from model compounds.

Reviewer #2 (Remarks to the Author):

In this very interesting and detailed paper, Y. Roman-Leshkov and G. T. Beckham challenge the commonly accepted (and not yet quantitatively supported) perception that higher S/G ratio is a reliable measure of monomer yield. In this work, the group has prepared poplars with varying S/G ratios and used RCF as analytical tool to determine monomer yields. Other fractions (involving important C-C bonds) were tracked and analysed. Interestingly, there was no correlation between S/G ratio and monomer yield. The authors proposed that the plant controls the amount of C-C bonds by regulating monomer transport across the cell wall during biosynthesis.
The research is timely and is of broader interest. The paper is rich in technical details and is of high

quality. I recommend publication after a few revisions listed below.
+++ For quantification of monomer yield as well as determination of the dimeric/oligomeric structures present – the catalytic reductive fractionation method was used. The logic behind this is obvious – this is currently the method of choice to deliver near-theoretical yields. However, imperfections of the method, some possible recondensation (or different degree of recondensation with different S/G ratio) cannot be excluded and RF is still a relatively new method. Also, the dimer structures were inferred by GC-MS (derivatization) but no fragmentation pattern analysis is given.
Thioacidolysis and derivatization followed by reductive cleavage (DFRC) is a known method that is established and contributed a lot to our understanding of lignin structure. (see for example ChemSusChem 2017, 10, 830.). A detailed analysis of the nature and distribution of the dimers was performed in this paper. Thioacidolysis /RC should be a good method to check the results against (upon non-destructive lignin isolation such as CEL).
Minor comments:
- Fig 3 (B) and the same for SI . Please add Mw, Mn, D values
- Fig 5 Please specify which experiments it is from (based on NMR, MS…etc), Fig 5 (A) which experiment (and time if important).
- Page 8 "…The consistent monomers yield between all of the different natural variants implies there may be some degree of control in the polymerization process"
Please specify what you mean by control.
- Please provide one example of how the NMR HSQC spectra were analyzed
-Page 1 "…synthesized from phenylalanine…" Should be "…from phenylalanyl and tyrosine…"
Ref Nat. Prod. Rep. 2008, 25, 1015.
Page 1 "…undergo a free radical polymerization…" Would it be better to rephrase to – lignin undergoes radical coupling rather than true radical polymerization?

Reviewer #3 (Remarks to the Author):

In this manuscript, the authors performed lignin hydrogenolysis on a dual-bed flow through reactor of natural poplar containing lignin with different S/G ratio ranging from 1.41 to 3.60. By analyzing the original S/G ratio, the distribution of S and G units in the produced lignin monomer and oligomer, and the yield of monomer and oil, they propose that the differences in S/G ratio of lignin in the plant do not predict the depolymerization monomer yields. Mainly, they point to the fact that they obtained higher monomer yields from the sample with the S/G ratio of 1.41 S/G compared to the sample with 3.60. I think this is an interesting result and the implications they propose for lignin biosynthesis could warrant publication in Nature Communications. However, I think the authors should limit their framing of this conclusion to the relatively limited scope of samples and S/G ratios that they studied (i.e. wild hardwood). Since they are using a homemade Ni catalyst, I also would like to see a few control experiments with commercial noble metal catalysts to rule out catalyst effects.

Major comments

1) The idea that lignin with higher S/G ratio produces higher monomer yield has largely been used to explain extreme case like the hydrogenolysis yields of softwood lignin (a lignin containing only G units, i.e., the S/G ratio is 0) and lignin with extremely high S content (for example transgenic poplar with an S/G ratio of 19). In contrast, they are dealing with samples with relatively small deviations. There is no doubt that the authors are presenting a provocative idea and have performed a careful study. However, with the data they present, the conclusion should be applied only to a fairly narrow range of S/G ratios in wild hardwood.
For the samples in between extreme S/G values, I disagree that the S/G ratio has been portrayed as the significant factor that is used to predict the depolymerization yield to monomers from lignin, which is an idea that the authors present throughout their manuscript. For example, in the abstract: "This study […] directly challenges the common conception that the S/G ratio is the sole metric to predict

monomer yields." I am not aware of any claims that this is the sole metric controlling yields. In fact, the main claim I have seen in the literature is that ether content controls yields and that in extreme cases S/G ratios can influence ether content. I think the authors' main result points to the fact that many factors are at play in controlling ether content (not just the nature of lignin subunits), especially for small variations in S/G ratios. However, they should be careful to avoid exaggerating the significance of their result especially for wider ranges of compositions.

2) Could the authors more accurately define their lignin yield definition? Also, it would be useful to be able to compare yields to those in the literature (the authors attempt to do this in Fig. S1). However, it seems that the authors are mixing yields determined based on Klason (work by Van den Bosh and Shuai), Klason + Acid soluble lignin (their work) and ABSL lignin (Parsell et al.). Perhaps they could (when possible) put yields on the same basis.

3) Along the same lines as comment 2, the authors obtained all their results with a homemade Ni/C catalyst. Could they replicate their most extreme results (lowest and highest S/G ratio) with commercial Ru/C or Pd/C in batch to be able to rule out any catalyst effect on their results? They could also compare this result to the same results obtained in literature (for example with Van den Bosh et al.) based on the same yield definition to benchmark the biomass with respect to previously obtained results.
4) Are any lignin monomers or oligomers being lost to the aqueous phase during the DCM-Water workup?
5) Table 1. Could the authors break the lignin in the compositional analysis into its Klason lignin and acid soluble lignin components?

Minor comments:
1) Please give the units for the extinction coefficient used during the compositional analysis of the acid soluble lignin.
2) Table S4 line 1 has a very high error for the total. I suspect a decimal point is missing
3) I found Table S1 a little hard to interpret. Could the authors present the mole fractions as percentages or decimals. Also, it could help to insert hashed lines between the different S/G ratios for added clarity. Could the authors give the sample time instead of sample number and provide a caption to describe the data and what it connects to?

Differences in S/G ratio in natural poplar variants do not predict catalytic depolymerization monomer yields: Implications for lignin biosynthesis

Eric M. Anderson[a],‡, Michael L. Stone[a],‡, Rui Katahira[b], Michelle Reed[b], Wellington Muchero[c],[d], Kelsey Ramirez[b], Gregg T. Beckham* [b],[d], Yuriy Román-Leshkov*,[a]

Review Response

Reviewers' comments:

Reviewer #1 (Remarks to the Author):

The manuscript by Roman-Leshkov, Beckham, and co-workers is an intriguing and original investigation on the influence of lignin's S/G ratio, its relation to lignin structure, and implications for lignin depolymerization. The authors exploit their recently developed flow-through RCF system as an analytical tool to study lignin depolymerization in function of reaction time. Surprisingly, the S/G ratio does not seem to correlate with lignin monomer yields. Rather the monomer concentration in the cell wall is presented as an important parameter that governs inter-unit bond distribution during lignin biosynthesis. In my opinion, these findings provide new insights, of general interest to the lignin community, and can be published in Nature communications. However, I feel the following revisions are required to support the conclusions.

• In my opinion, presenting the phenolic distribution as S/G-ratio gives the reader an (erroneous) impression that the distribution differences are very large. Presenting the phenolic distribution as %S and %G is more appropriate in my opinion, and shows that the S-content only differs between 58% and 78% of the total phenolics.

We have modified table 1 in the main text to include the phenolic distribution in terms of %S, and edited the text to explicitly reference how S/G ratio relates to %S content:

"In this study, we used RCF to investigate the impact of the S/G ratio on the production of monomers using natural poplar variants with S/G contents ranging from 1.41 to 3.60, or percent S content of 58.5% to 78.3% (Table 1)"

Table 1: Compositional analysis of poplar natural genetic variants.

S/G	% S	% Lignin	% Glucan	% Xylan	% Galactan	% Arabinan	% Mannan	% Acetyl	% Total
1.41	58.5	27.1	39.4	16.8	1.7	0	5.2	4.2	96.7
1.69	62.8	25.1	42.4	14.6	1.8	0	4.4	3.7	95.1
2.35	70.1	23.9	44.7	15.0	1.9	0	5.3	3.9	96.8
3.48	77.7	25.0	39.7	17.3	1.7	0	5.2	4.1	95.9
3.60	78.3	22.6	42.7	17.2	1.8	0	5.1	4.0	96.0

• To strengthen the conclusion, it would be good if the authors could investigate a larger variation in the phenolic distribution, and maybe a larger sample set. Sample 4 and 5 have the same %S.

While we agree with the reviewer that a larger variation in the phenolic distribution would be advantageous, the samples we chose span the range of S/G ratios available within the naturally variant population. We have included in the response a figure from the paper "Lignin content in natural *Populus* variants affects sugar release" (Studer et al. PNAS 2011), which shows the S/G ratios of poplar trees across the entire GWAS population as determined by pyrolysis MBMS. We chose to limit this study to five samples to enable in depth characterization of each sample, and chose to include S/G ratios at the extremes of the natural variant range.

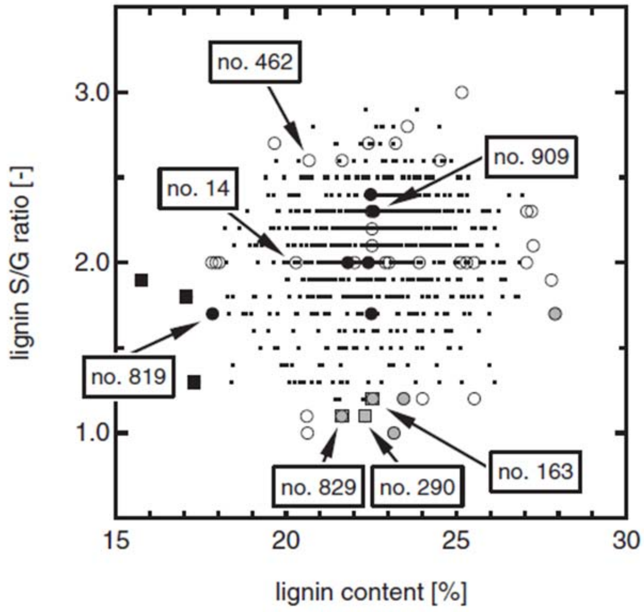

The following sentence has been added to the introduction:

In this study, we used RCF to investigate the impact of the S/G ratio on the production of monomers using natural poplar variants with S/G contents ranging from 1.41 to 3.60, or percent S content of 58.5% to 78.3% (Table 1).[31] These 5 samples capture the range of S/G ratios present in a naturally variant population of over 1000 poplar trees.[32]

• I appreciate the approach of using RCF as an analytical technique, though I strongly suggest to benchmark the results with a generally accepted analytical technique, for instance thioacidolysis.

We have performed thioacidolysis on the 5 samples and have included the results in the revised Supporting Information. Please see the response to the next comment for the in-depth discussion and manuscript modifications.

• The authors mention in the discussion that incomplete depolymerization nor recondensation can explain the observed trends. Considering recondensation, I feel that this should be demonstrated experimentally and not merely based on literature. The absence of condensation could be demonstrated by showing that other (noble metal) catalysts result in similar monomer yields and distributions (for example in batch runs). This would demonstrate that the obtained yield is indeed a theoretical maximum yield.

The following paragraph has been added to the results section:

Furthermore, to confirm that the lack of correlation between monomer yields and the S/G ratio was not caused by the RCF method or by limitations due to catalyst choice, we performed batch RCF with a commercial ruthenium on carbon (Ru/C) catalyst as well as thioacidolysis on each sample. Thioacidolysis is a common lignin depolymerization

technique used to measure the relative content of lignin monomers bound by β-O-4 linkages.[45-47] These data, summarized in Tables S2-3 and Figures S3-4 show that neither RCF with Ru/C or thioacidolysis yields correlate with S/G ratio. An important metric to directly compare the depolymerization efficiency of Ru/C with Ni/C is the monomer/oil ratio (i.e. the weight fraction of the lignin oil made up by monomers). While Ru/C generated slightly higher monomer/oil ratios than Ni/C, it showed no trend in monomer/oil ratio across the S/G range. These experiments confirmed that the original conclusions drawn from the Ni/C RCF results were valid, despite slight variations in results between different depolymerization methods, and validate the use of flow-through RCF as an analytical technique.

Also, a sentence was added to the discussion:

Shuai et al. also showed that monomer yields from a formaldehyde-stabilized lignin were identical to those produced from direct RCF of the same biomass. In this work, we demonstrated similar trends using either Ni/C or commercial Ru/C catalysts, indicating the results obtained were not caused by the type of catalyst used. Therefore, the supercritical RCF experiments performed within this study should indicate a true monomer yield for each natural variant, ruling out re-condensation as the cause of the observed trends.

Table S2: Thioacidolysis yields for biomass samples. Standard deviation for n=2 reaction replicates shown in parenthesis. Thioacidolysis was performed on 2 mg of ground sample, as reported in Harman-Ware et al.[3]

S/G by Py-MBMS	H (µmol/g sample)	S (µmol/g sample)	G (µmol/g sample)	Total Yield (µmol/g sample)	Measured S/G
1.41	5.7 (±0.0)	330.2 (±6.5)	138.8 (±1.6)	474.7 (±4.9)	2.4 (±0.1)
1.69	5.9 (±0.0)	260.5 (±0.8)	125.9 (±4.5)	392.3 (±5.3)	2.1 (±0.1)
2.35	6.1 (±0.0)	299.0 (±13.0)	106.6 (±1.9)	411.7 (±14.9)	2.8 (±0.1)
3.48	4.8 (±0.0)	353.5 (±9.7)	91.3 (±0.1)	449.6 (±9.9)	3.9 (±0.1)
3.6	5.7 (±0.1)	325.6 (±22.1)	89.5 (±6.8)	420.8 (±29.0)	3.6 (±0.0)

Table S3: Comparison of Thioacidolysis and batch RCF yields. Yields are all shown on the same basis – micro-mole of monomer per gram of biomass sample loaded. Measured S/G was calculated based on a molar ratio of monomeric S and G units. The Batch RCF results are the same as those shown in Figure S3.

	Ni/C Supercritical Batch RCF Results		Ru/C Supercritical Batch RCF Results		Thioacidolysis Results	
S/G by Py-MBMS	Monomer Yield (µmol/g sample)	Measured S/G	Monomer Yield (µmol/g sample)	Measured S/G	Monomer Yield (µmol/g sample)	Measured S/G
1.41	469.7	1.9	453.7	1.8	474.7 (±4.9)	2.4 (±0.1)
1.69	402.4	1.6	429.6	1.6	392.3 (±5.3)	2.1 (±0.1)
2.35	393.9	2.1	429.7	2.1	411.7 (±14.9)	2.8 (±0.1)
3.48	390.8	2.7	445.1	2.7	449.6 (±9.9)	3.9 (±0.1)
3.6	334.5	2.7	418.4	2.8	420.8 (±29.0)	3.6 (±0.0)

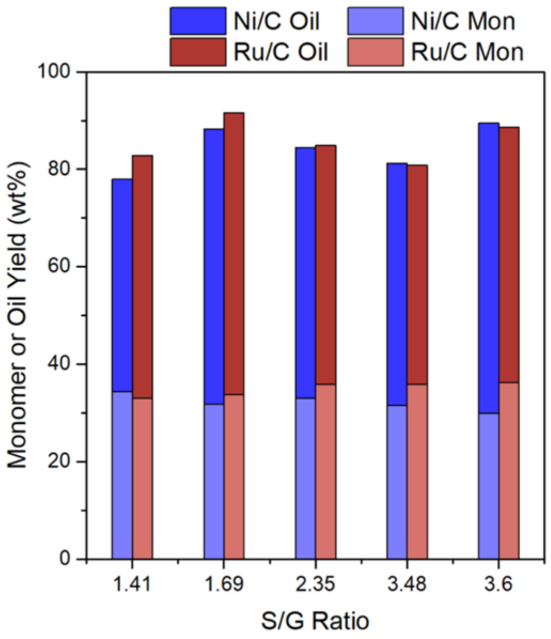

Figure S3. Comparison of batch RCF results using Ni/C and Ru/C catalysts. Standard deviations of 1.66% and 0.98% were calculated for the monomer yield and the oil yield, respectively, based on a replicate using Ru/C on the S/G = 3.6 sample. Yields were calculated relative to initial lignin content present in the biomass (Klason + acid soluble). Reaction conditions: 0.96-1.15 g of poplar wood (0.26g lignin), 0.15 g 15 wt% Ni/C or 0.2 g 5 wt% Ru/C, 50 mL MeOH, 250°C, 700 RPM and 30 bar of H_2 (STP).

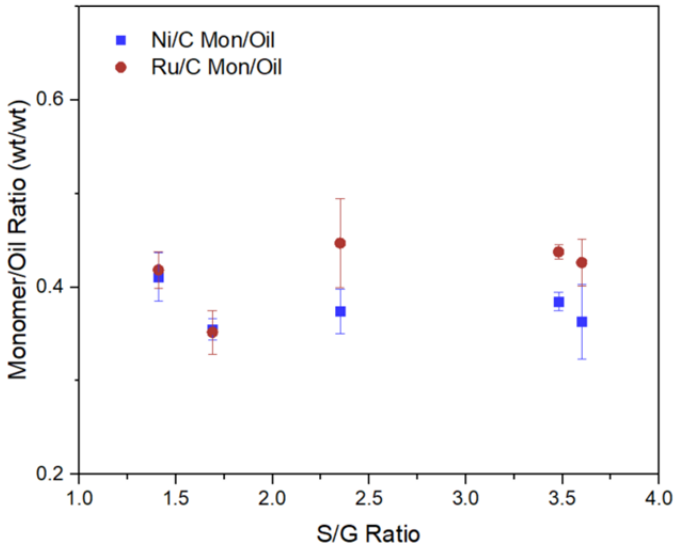

Figure S4. Comparison of batch RCF results using Ni/C and Ru/C catalysts. Monomer/oil ratios were calculated as the ratio of the total lignin monomer yield to the total lignin oil yield on a weight basis. Error bars were generated using replicates performed on a scaled-down batch reactor system, using identical reaction conditions to those shown in Figure S2 with loadings downscaled to 3/5 of the original.

For reference, the tabulated data used to generate Figure S3 and S4 is shown below:

S/G	0.15 g of 15 wt% Ni/C			0.2 g of 5 wt% Ru/C		
	Monomer Yield	Oil Yield	Mon/Oil	Monomer Yield	Oil Yield	Mon/Oil
1.41	34.37	78.02	0.441	32.97	82.86	0.40
1.69	31.81	88.29	0.360	33.72	91.59	0.37
2.35	33.04	84.49	0.391	35.86	84.89	0.422
3.48	31.58	81.26	0.389	35.85	80.85	0.443
3.6	29.96	89.46	0.335	36.17	88.64	0.408

The table below shows additional data generated using a smaller batch system used to obtain error bars (standard deviation calculated with these data and the data above) on the monomer to oil yield ratios (shown in figure S4). The same biomass/catalyst/methanol ratios were utilized. All values were downscaled to 3/5 of the original values. Although at this smaller scale product losses during transfer and extraction made up a larger percentage of the yield (resulting in lower monomer and oil yield values), the monomer/oil ratios were comparable to those obtained in the larger batch experiments. Indeed, the monomer/oil ratio is a measure of depolymerization efficiency that is independent of the total oil recovery. The monomer/oil ratio is a more important metric when assessing the catalytic activity and the variation between biomass samples, as this is independent of the extent of extraction. The comparison between Ni/C and Ru/C shows some slight differences, but neither catalyst shows a trend with S/G ratio.

S/G	0.15 g of 15 wt% Ni/C				0.2 g of 5 wt% Ru/C			
	Monomer Yield	Oil Yield	Mon/Oil	Mon/Oil Error Bar	Monomer Yield	Oil Yield	Mon/Oil	Mon/Oil Error Bar
1.41	27.21	68.32	0.398	0.02596	29.56	67.75	0.44	0.01939
	28.71	73.00	0.393		26.47	62.83	0.42	
1.69	27.20	74.96	0.363	0.01166	23.14	68.97	0.34	0.02315
	26.11	76.47	0.341					
2.35	27.62	77.23	0.358	0.02366	32.69	65.16	0.502	0.04750
					32.12	77.07	0.417	
3.48	27.02	68.92	0.392	0.00987	30.01	69.46	0.432	0.00802
	28.65	76.71	0.373					
3.6	29.19	71.35	0.409	0.03995	28.08	62.35	0.450	0.02531
	28.74	82.98	0.346		31.612	72.103	0.438	

• The conclusions seem to align with conclusions from ref. 26. I suggest the authors to include this in the discussion.

We thank the reviewer for bringing this useful point to our attention and have added a sentence in the discussion of figure 6 (on the relative formation of B-B and B-O-4 bonds with varying lignin concentrations):

"This concept, illustrated in Figure 6, was also shown experimentally by Stewart et al., who found that genetically modified poplar with 97.5% S showed a significant increase in levels of β-β linkages (relative to native S-levels in wild-type trees) but an insignificant increase in β-ether levels.[27]"

• Related to the previous comment, I feel the following paper should be included/discussed. van Parijs FR, Morreel K, Ralph J, Boerjan W, Merks RM. Modeling lignin polymerization. I. Simulation model of dehydrogenation polymers. Plant Physiol. 2010;153(3):1332-44. Herein, the flux of monomers during lignin biosynthesis is also appointed as an important factor in the distribution between C-C and C-O-C interunit linkages, though the results are based on model insights in combination with data from model compounds.

We thank the reviewer for bringing up another useful reference, and have added a sentence into the discussion:

Furthermore, when a dialysis membrane was used with both sinapyl and coniferyl alcohol, an insoluble polymer was generated exhibiting similar properties to lignin.[53-54] The importance of monomer delivery rate was shown computationally by van Parijs et al. who demonstrated that decreasing the influx of monolignols in a lignin polymerization model increased the amount of β-O-4 formation by favoring the growth of longer chains and decreasing dimerization.[55] Thus, during lignin biosynthesis, it is plausible that high sinapyl alcohol concentrations relative to the number of growing chains will lead to β-β formation, while a low sinapyl alcohol concentration during lignification would likely lead to formation of β-O-4 in a strictly syringyl polymer

Reviewer #2 (Remarks to the Author):

In this very interesting and detailed paper, Y. Roman-Leshkov and G. T. Beckham challenge the commonly accepted (and not yet quantitatively supported) perception that higher S/G ratio is a reliable measure of monomer yield. In this work, the group has prepared poplars with varying S/G ratios and used RCF as analytical tool to determine monomer yields. Other fractions (involving important C-C bonds) were tracked and analysed. Interestingly, there was no correlation between S/G ratio and monomer yield. The authors proposed that the plant controls the amount of C-C bonds by regulating monomer transport across the cell wall during biosynthesis.
The research is timely and is of broader interest. The paper is rich in technical details and is of high quality. I recommend publication after a few revisions listed below.

+++ For quantification of monomer yield as well as determination of the dimeric/oligomeric structures present – the catalytic reductive fractionation method was used. The logic behind this is obvious – this is currently the method of choice to deliver near-theoretical yields. However, imperfections of the method, some possible recondensation (or different degree of recondensation with different S/G ratio) cannot be excluded and RF is still a relatively new method. Also, the dimer structures were inferred by GC-MS (derivatization) but no fragmentation pattern analysis is given.

Please see additional data and discussion in response to comment 4 from reviewer 1. Briefly, we performed additional experiments involving thioacidolysis as well as RCF with commercial Ru/C catalyst. Across all techniques we observed similar yields and no trends with S/G ratio.

All MS fragmentation patterns are included in part 5 of the supplemental information. In the revised version, we split part 5 into 2 sections: newly identified dimers and dimers previously identified in the literature. We have now added fragmentation analysis showing ions corresponding to major fragments for the newly identified dimers shown in this work. We have also included literature references for the rest of the previously identified dimers in part 5.2.

Thioacidolysis and derivatization followed by reductive cleavage (DFRC) is a known method that is established and contributed a lot to our understanding of lignin structure. (see for example ChemSusChem 2017, 10, 830.). A detailed analysis of the nature and distribution of the dimers was performed in this paper. Thioacidolysis /RC should be a good method to check the results against (upon non-destructive lignin isolation such as CEL).

We have performed thioacidolysis on the poplar samples and included the data in the supporting information. Please see the data and discussion in response to comment 4 from reviewer 1. Additionally, we thank the reviewer for bringing this reference to our attention and have included it in our discussion of the thioacidolysis results.

Minor comments:
- Fig 3 (B) and the same for SI . Please add Mw, Mn, D values

The following tables have been added in the SI

Table S3: Mn, Mw, and PD calculated from GPC results (main text figure 3B) for each lignin oil generated in flow-through RCF for each poplar sample at 1 h, 2-3 h and 4-6 h on stream.

S/G Ratio	Time on Stream	Mn	Mw	PD
1.41	1 h	332	448	1.3494
	2-3 h	377	554	1.4695
	4-6 h	406	622	1.532
1.69	1 h	341	476	1.3959
	2-3 h	390	597	1.5308
	4-6 h	418	669	1.6005
2.35	1 h	335	475	1.4179
	2-3 h	378	575	1.5212
	4-6 h	398	620	1.5578
3.48	1 h	314	416	1.3248
	2-3 h	358	543	1.5168
	4-6 h	378	589	1.5582
3.6	1 h	322	442	1.3727
	2-3 h	376	588	1.5638
	4-6 h	402	639	1.5896

Table S9: Mn, Mw, and PD calculated from GPC results (Figure S10) for each lignin oil generated in Batch RCF.

S/G Ratio	Mn	Mw	PD
1.41	321	450	1.4019
1.69	331	478	1.4441
2.35	322	448	1.3913
3.48	300	419	1.3967
3.6	313	438	1.3994

- Fig 5 Please specify which experiments it is from (based on NMR, MS…etc), Fig 5 (A) which experiment (and time if important).

A more detailed description was included in the discussion of the dimer analysis:

"To study this, the lignin oils from the same five flow-through RCF experiments at 1 h, 2-3 h and 4-6 h on stream were silylated to increase the volatility of the dimeric lignin fragments in the oil"

The figure 5 caption was also expanded:

"Figure 1: Distribution of C-C linked dimers observed from flow-through RCF reactions (same samples as Figures 2 and 3). (A) Chromatogram from a representative GC-MS run with a silylated lignin oil (specifically, S/G=1.69 at 1 h on stream). The intensity of the peaks measured in total ion counts. (B) The relative occurrence of the different coupling partners at different times on stream as determined by relative GC-MS total ion count peak areas. (C) The relative distribution of linkage types with in each monomers coupling pair as determined by relative GC-MS total ion count peak areas."

- Page 8 "…The consistent monomers yield between all of the different natural variants implies there may be some degree of control in the polymerization process"
Please specify what you mean by control.

We have edited the text to be more specific:

"The consistent monomer yields between all of the different natural variants implies there may be some degree of control exerted by the plant that influences the types of linkages formed in the polymerization process"

- Please provide one example of how the NMR HSQC spectra were analyzed

The following sentence was added in the NMR discussion in the main text:

An example of the HSQC NMR analysis is shown in the Supplemental Information, Figure S5.

And, the following figure and discussion were added to the SI:

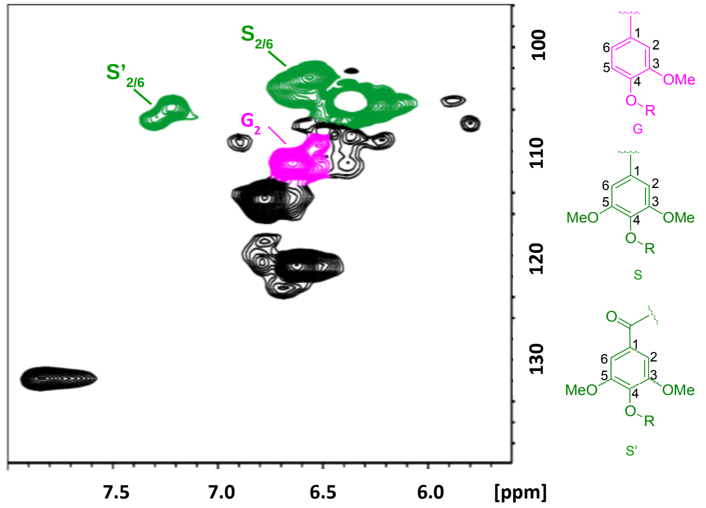

Figure S5. Expanded HSQC NMR spectrum of lignin oil from S/G = 2.35 at 2-3 h on stream in the aromatic region. G: guaiacyl, S: syringyl, and S': syringyl with Cα=O

Integration of each peak is 2676 of S2/6, 50 of S'2/6 and 385 of G2. Total integral of S2/6 and S'2/6 corresponded to two correlations, while integral of G2 corresponded to one correlation. Therefore, based on the integration values, S/G ratio was calculated as follows.
{(S2/6 + S'2/6)/2}/G2 = {(2676 + 50)/2}/385 = 3.54

-Page 1 "…synthesized from phenylalanine…" Should be "…from phenylalanyl and tyrosine…" Ref Nat. Prod. Rep. 2008, 25, 1015.

This has been corrected in the text and the reference has been added:

"synthesized from phenylalanyl and tyrosine in the cytoplasm"

Page 1 "…undergo a free radical polymerization…" Would it be better to rephrase to – lignin undergoes radical coupling rather than true radical polymerization?

This has been changed in the text:

"The plant transports monomers to the cell wall where they undergo free radical coupling reactions creating a variety of C-O and C-C linkages"

Reviewer #3 (Remarks to the Author):

In this manuscript, the authors performed lignin hydrogenolysis on a dual-bed flow through reactor of natural poplar containing lignin with different S/G ratio ranging from 1.41 to 3.60. By analyzing the original S/G ratio, the distribution of S and G units in the produced lignin monomer and oligomer, and the yield of monomer and oil, they propose that the differences in S/G ratio of lignin in the plant do not

predict the depolymerization monomer yields. Mainly, they point to the fact that they obtained higher monomer yields from the sample with the S/G ratio of 1.41 S/G compared to the sample with 3.60. I think this is an interesting result and the implications they propose for lignin biosynthesis could warrant publication in Nature Communications. However, I think the authors should limit their framing of this conclusion to the relatively limited scope of samples and S/G ratios that they studied (i.e. wild hardwood). Since they are using a homemade Ni catalyst, I also would like to see a few control experiments with commercial noble metal catalysts to rule out catalyst effects.

Major comments

1) The idea that lignin with higher S/G ratio produces higher monomer yield has largely been used to explain extreme case like the hydrogenolysis yields of softwood lignin (a lignin containing only G units, i.e., the S/G ratio is 0) and lignin with extremely high S content (for example transgenic poplar with an S/G ratio of 19). In contrast, they are dealing with samples with relatively small deviations. There is no doubt that the authors are presenting a provocative idea and have performed a careful study. However, with the data they present, the conclusion should be applied only to a fairly narrow range of S/G ratios in wild hardwood.
For the samples in between extreme S/G values, I disagree that the S/G ratio has been portrayed as the significant factor that is used to predict the depolymerization yield to monomers from lignin, which is an idea that the authors present throughout their manuscript. For example, in the abstract: "This study […] directly challenges the common conception that the S/G ratio is the sole metric to predict monomer yields." I am not aware of any claims that this is the sole metric controlling yields. In fact, the main claim I have seen in the literature is that ether content controls yields and that in extreme cases S/G ratios can influence ether content. I think the authors' main result points to the fact that many factors are at play in controlling ether content (not just the nature of lignin subunits), especially for small variations in S/G ratios. However, they should be careful to avoid exaggerating the significance of their result especially for wider ranges of compositions.

We agree with this point from reviewer 3, and the comment that the "main result points to the fact that many factors are at play in controlling ether content (not just the nature of lignin subunits)" is exactly the conclusion we are attempting to show. We appreciate this comment and have made some editorial changes accordingly. However, we would like to point out that the samples used represent a very wide range of S/G ratios for naturally variant plants within the same species. Please see our response to comment 2 from reviewer 1 for a distribution of the larger population and a discussion of our choice of samples.

In the abstract, the sentence referenced by reviewer 3 now reads: "directly challenges the common conception that the S/G ratio predicts monomer yields"

In the introduction: "Indeed, a higher S/G ratio in lignin has been shown to produce higher monomer yields using reductive catalytic fractionation (RCF) as a depolymerization method, as can be seen in Figure S1, where we compiled a variety of monomer yields found in the literature for across a vast range of S/G ratios from different natural and genetically modified feedstocks."

In the discussion: The results of study contained herein show that there is no correlation between S/G ratio and monomer yields within the range of S/G ratios in the naturally variant poplar population.

2) Could the authors more accurately define their lignin yield definition? Also, it would be useful to be able to compare yields to those in the literature (the authors attempt to do this in Fig. S1). However, it seems that the authors are mixing yields determined based on Klason (work by Van den Bosh and Shuai), Klason + Acid soluble lignin (their work) and ABSL lignin (Parsell et al.). Perhaps they could (when possible) put yields on the same basis.

We have more explicitly stated the lignin yield calculation in the text:

"At 6 h on stream, the cumulative monomer yield was approximately 23 wt% from samples with an S/G of 1.41, 2.35, 3.48, and 3.60 while the one with an S/G of 1.69 produced a monomer yield of 20 wt% (Figure 2A). The yields were calculated relative to total lignin content in the biomass initially, defined as the sum of Klason and acid-soluble lignin content."

And in the caption for figure 2:

"…A) Cumulative monomer yields for all five poplar natural variants (relative to Klason + acid-soluble lignin in biomass initially)…"

The direct calculation was added to the SI to clarify.

$$Monomer\ yield = \frac{mass\ of\ monomers}{(wt\%\ Klason + wt\%ASL) * mass\ of\ dry\ biomass}$$

3) Along the same lines as comment 2, the authors obtained all their results with a homemade Ni/C catalyst. Could they replicate their most extreme results (lowest and highest S/G ratio) with commercial Ru/C or Pd/C in batch to be able to rule out any catalyst effect on their results? They could also compare this result to the same results obtained in literature (for example with Van den Bosh et al.) based on the same yield definition to benchmark the biomass with respect to previously obtained results.

Please see the response to comment 4 from reviewer 1. Briefly, we have included results with commercial Ru/C in addition to thioacidolysis to rule out process and catalyst effect on our results. We came to the conclusion that despite minor differences between the methods, there is still no strong correlation between monomer yields and S/G ratio.

4) Are any lignin monomers or oligomers being lost to the aqueous phase during the DCM-Water workup?

We did not observe significant losses during work-up as shown in Figure S2 below.

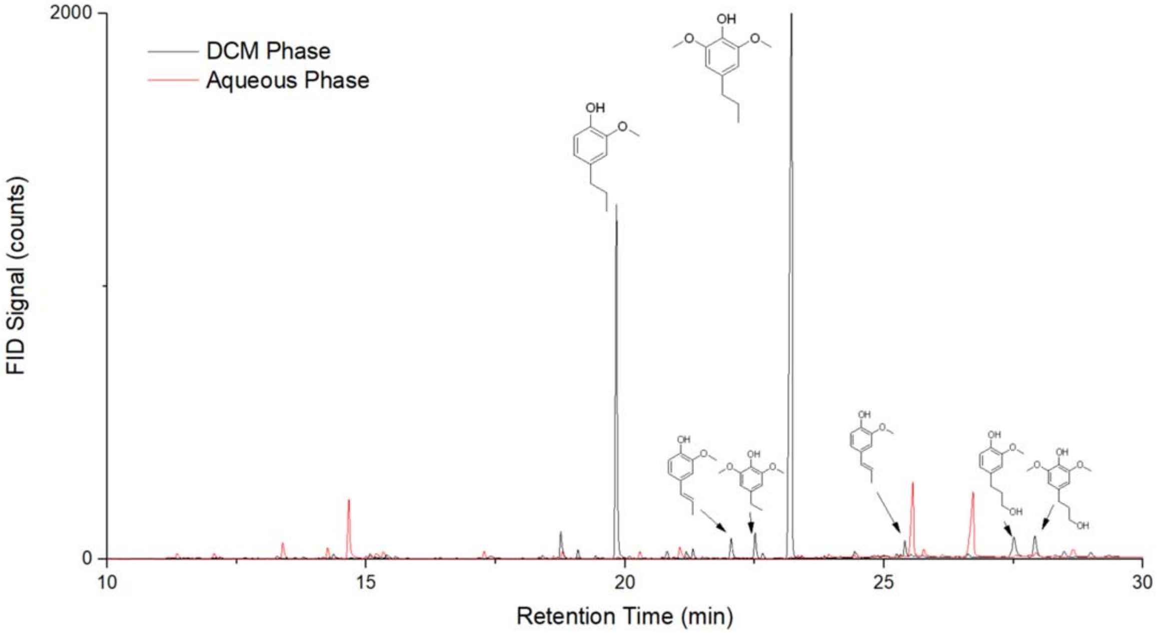

Figure S2. GC-FID was performed on the aqueous phase after DCM extraction on a supercritical batch RCF experiment with Ru/C. The minor peaks corresponding to lignin monomer compounds in the aqueous phase correspond to ~1.3% monomer yield (versus 32% in the DCM phase)

The following sentence was added in the results section: The resulting oil containing both monomers and oligomers, defined as lignin oil, was used as a metric to describe the extent of lignin extraction. Minimal losses of lignin products during DCM extraction were confirmed using GC-FID analysis of the aqueous phase (Figure S2).

5) Table 1. Could the authors break the lignin in the compositional analysis into its Klason lignin and acid soluble lignin components?

The lignin content was broken down into both Klason and acid soluble (ASL) lignin components. The flow through studies were performed such that the total lignin (ASL+Klason) were kept constant between runs. Therefore, all samples contain were run with varying amounts of Klason lignin. This table was added to the SI as Table S1.

S/G	Klason Lignin (wt%)	ASL (wt%)	Total Lignin (wt%)
1.41	22.8±1.2	4.3±0.5	27.1±1.4
1.69	20.9±0.9	4.2±0.2	25.1±0.7
2.35	19.3±0.2	4.6±0.2	23.9±0.1
3.48	19.7±0.4	5.3±0.5	25.0±0.9
3.60	17.3±1.5	5.3±0.4	22.6±1.3

Minor comments:

1) Please give the units for the extinction coefficient used during the compositional analysis of the acid soluble lignin.

The extinction coefficient used for the UV determination of acid soluble lignin is listed in the SI in the section on biomass compositional analysis.

"The remaining acid soluble lignin was determined by UV/Vis spectroscopy (Thermo Scientific Nanodrop 8000 spectrophotometer), using absorbance measurements at 240 nm with an extinction coefficient of 2.5."

2) Table S4 line 1 has a very high error for the total. I suspect a decimal point is missing

We thank the reviewer for noticing this typo and have corrected it. The correct error is 1.5 and not 15.

3) I found Table S1 a little hard to interpret. Could the authors present the mole fractions as percentages or decimals. Also, it could help to insert hashed lines between the different S/G ratios for added clarity. Could the authors give the sample time instead of sample number and provide a caption to describe the data and what it connects to?

The table and caption were modified to include more detail as follows:

Table S5: Mole fractions of S in the oligomer fraction of oils generated in flow-through RCF of poplar natural variants. These values were calculated using equations S.4 and S.5, and S/G ratios generated from integration of HSQC NMR peaks. The data correspond to 1 h, 2-3 h and 4-6 h combined oil samples from reactions performed with the following conditions: 0.96-1.15 g of poplar wood (0.26 g lignin), 0.3 g of 15% Ni/C (50/50 SiO_2), 190^oC both beds, 0.5 mL min^{-1} MeOH, 50 mL min^{-1} H_2 at 60 bar.

S/G	1.41		1.69		2.35		3.48		3.6	
Time range	x_S	x_G	x_S	x_G	x_S	x_G	x_S	x_G	x_S	x_G
1 h	43	35	47	38	46	39	48	36	47	36
2-3 h	56	49	62	56	56	47	54	69	59	52
4-6 h	69	66	74	67	69	66	70	61	69	60
Average	58	49	63	54	57	48	58	60	60	49

-

REVIEWERS' COMMENTS:

Reviewer #1 (Remarks to the Author):

The authors did a very good job in revising their manuscript, and explained their changes in a detailed rebuttal letter. As I previously mentioned, the results and conclusions of this manuscript are of general interest to the lignin community, and can thus be published in Nature communications. The authors might want to look at some minor suggestions listed below:

- Fig 2. in the main text: R8 & R9 (in beta-beta III) are not defined.
- Fig. S6 in the supporting information: I had a hard time trying to interpret this figure, due to the complex symbol definitions. I feel there is to much information in one graph.
- Fig. S3 in the supporting information. The monomer yields with Ru/C differ quite a bit from the results from Ni/C, especially for the high S/G ratio's. Moreover, it is mentioned that Ru/C RCF yields do not correlate with S/G ratio. I'm not absolutely sure, there seems to be some sort of correlation, though a very weak correlation?

Reviewer #2 (Remarks to the Author):

The authors have addressed all points raised by the referees - i recommend the paper to be published.

Reviewer #3 (Remarks to the Author):

The authors did a very good job of addressing my comments. Publish without further revisions.

Differences in S/G ratio in natural poplar variants do not predict catalytic depolymerization monomer yields

Eric M. Anderson[a],‡, Michael L. Stone[a],‡, Rui Katahira[b], Michelle Reed[b], Wellington Muchero[c],[d], Kelsey Ramirez[b], Gregg T. Beckham* [b],[d], Yuriy Román-Leshkov*,[a]

Review Response

REVIEWERS' COMMENTS:

Reviewer #1 (Remarks to the Author):

The authors did a very good job in revising their manuscript, and explained their changes in a detailed rebuttal letter. As I previously mentioned, the results and conclusions of this manuscript are of general interest to the lignin community, and can thus be published in Nature communications. The authors might want to look at some minor suggestions listed below:

- Fig 2. in the main text: R8 & R9 (in beta-beta III) are not defined.

Figure 2 has been modified. We thank the reviewer for noticing this.

- Fig. S6 in the supporting information: I had a hard time trying to interpret this figure, due to the complex symbol definitions. I feel there is to much information in one graph.

We have included a clarifying statement to the figure caption, to help guide the reader to understand the general point we are hoping to prove.

"*Supplementary Figure 7: Oligomer mole fractions calculated from the mole balance determined from monomer yields and HSQC NMR. The mole fraction displays how much of S units or G units are contained in C-C bonds in the lignin extracted from flow-through RCF experiments. Generally, it can be seen that more S-units (closed shapes) are partitioning into the oligomer fraction than G-units (open shapes) are partitioning into the oligomer fraction. This is true for nearly all time points and nearly all biomass samples.*"

- Fig. S3 in the supporting information. The monomer yields with Ru/C differ quite a bit from the results from Ni/C, especially for the high S/G ratio's. Moreover, it is mentioned that Ru/C RCF yields do not correlate with S/G ratio. I'm not absolutely sure, there seems to be some sort of correlation, though a very weak correlation?

While the overall monomer yields may show a very weak correlation, the monomer yields (normalized to the mass of lignin loaded into the batch reactor) are dependent on both the lignin structure (i.e. distribution and amount of cleavable ether bonds) and the extent of lignin extraction. To isolate the effect of lignin structure from the extent of lignin extraction, we plotted the monomer/oil ratio. In this case, we saw no correlation between S/G ratio and depolymerization efficiency with either Ru/C or Ni/C.

Reviewer #2 (Remarks to the Author):

The authors have addressed all points raised by the referees - i recommend the paper to be published.

Reviewer #3 (Remarks to the Author):

The authors did a very good job of addressing my comments. Publish without further revisions.
